# History matching through dynamic decision-making

**DOI:** 10.1371/journal.pone.0178507

**Published:** 2017-06-05

**Authors:** Cristina C. B. Cavalcante, Célio Maschio, Antonio Alberto Santos, Denis Schiozer, Anderson Rocha

**Affiliations:** 1 Institute of Computing, University of Campinas, Campinas, São Paulo, Brazil; 2 School of Mechanical Engineering, Center for Petroleum Studies, University of Campinas, Campinas, São Paulo, Brazil; IUMPA - Universitat Politecnica de Valencia, SPAIN

## Abstract

History matching is the process of modifying the uncertain attributes of a reservoir model to reproduce the real reservoir performance. It is a classical reservoir engineering problem and plays an important role in reservoir management since the resulting models are used to support decisions in other tasks such as economic analysis and production strategy. This work introduces a dynamic decision-making optimization framework for history matching problems in which new models are generated based on, and guided by, the dynamic analysis of the data of available solutions. The optimization framework follows a ‘learning-from-data’ approach, and includes two optimizer components that use machine learning techniques, such as unsupervised learning and statistical analysis, to uncover patterns of input attributes that lead to good output responses. These patterns are used to support the decision-making process while generating new, and better, history matched solutions. The proposed framework is applied to a benchmark model (UNISIM-I-H) based on the Namorado field in Brazil. Results show the potential the dynamic decision-making optimization framework has for improving the quality of history matching solutions using a substantial smaller number of simulations when compared with a previous work on the same benchmark.

## 1 Introduction

In the context of management of petroleum fields, numeric reservoir models aim at representing a real reservoir system and allow forecasting the reservoir performance in different production stages and under different operating conditions.

The construction of a reservoir model is a multidisciplinary task and involves contributions from different areas such as seismic, petrophysics, geology and field engineering. Due to the complex nature of reservoir systems, the model characterization has to cope with many uncertainties (for example, in the estimates of the reservoir rock and fluid properties). As a consequence, the initial best estimate reservoir model is very likely inadequate to obtain an accurate prediction of the real reservoir performance. Handling uncertain attributes so that an initial reservoir model can be calibrated to reproduce real reservoir performance is the purpose of a *history matching* process. The goal is to get better calibrated reservoir models, with simulation results closer to the observed data, so that future reservoir predictions based on these models are more reliable and accurate. History matching plays an important role in reservoir management since the resulting models are used to support the decision-making process in other tasks such as economic analysis and production strategy. A walk-through example of the basic concepts of the history matching problem can be found in S1 Appendix.

Formally speaking, history matching can be defined as the process of finding combinations of the reservoir model uncertain attributes which minimize the difference between the simulated data and the observed data. It is a typical inverse problem where the output is known (the observed reservoir dynamic data) and one needs to find the input parameters (the values of the reservoir model uncertain attributes) which leads to the observed data.

The complexity of the history matching process is directly related to the number of reservoir model uncertain attributes considered during the process and the output variables that need to be matched. Also, the nature of the reservoir models is such that, frequently, there are multiple equivalent solutions for a history matching problem.

The evaluation of each solution for a history matching problem normally requires a costly simulation. For this reason, finding efficient strategies that reduce the number of simulations required to achieve good solutions is a continuous challenge of the area.

The literature on computer-assisted history matching is extensive. A review on the recent progress on the area can be found in [1].

Among the algorithmic approaches, stochastic optimization methods are the ones which have mostly been used. Mohamed et al [2], for example, provided a comparison of three of such methods, Hamiltonian Monte Carlo (HMC) algorithm, Particle Swarm Optimization (PSO) algorithm, and the Neighborhood Algorithm (NA), when used to generate multiple history matched models. Hajizadeh et al. [3] applied an Ant Colony Optimization (ACO) algorithm to find history matching solutions for two reservoir simulation cases. Maschio and Schiozer [4] proposed a Bayesian history matching method using an iterative procedure that combines Markov Chain Monte Carlo (MCMC) sampling with an artificial neural network (ANN) proxy for the numerical simulator.

Geostatistical history matching is another approach which is being increasingly applied. Caeiro et al. [5], for example, proposed a methodology that combines a global optimization stage, over geological parameters, with a refining optimization stage based on individual well production matches. A framework to perform history matching integrated with geostatistical modeling was proposed by Maschio et al. [6], where a genetic algorithm was used to redefine the bounds of geological parameters and reduce the search space during the optimization process.

Efforts in the development of methodologies that combine history matching with uncertainties reduction can also be identified. Maschio and Schiozer [7] recently presented a framework to reduce uncertainties, where a genetic algorithm was used to search in a space formed by the parameters of the probability distribution functions of the uncertain attributes. In addition, in their work, artificial neural networks were used, instead of the numerical simulator, to model the curves of the output variables. Another example of history matching integrated with uncertainties reduction can be found in the work of Mesquita et al. [8], which proposed indicators to quantify the matching quality and to support the identification of input attributes which may be source of high output deviations. The proposed indicators were also used to define guidelines of possible actions aiming at reducing the uncertainties and to improve the history matching.

The many and different approaches to solve history matching problems are justified because, given specific characteristics of each reservoir systems, there is no strategy that is proven effective for all cases. Stochastic methods, for example, are easy to implement. However, depending on the complexity of the history matching problem, the application of such methods can be computationally prohibitive, since a high number of simulations may be required to reach convergence. The use of proxy models to replace the flow simulator allows fast evaluation of the history matching objective function, what facilitates the exploration of the search space. The disadvantage of such approach is that generate accurate proxies is a hard task in the context of non-linear problems, as is the case of history matching.

Also, considering that each history matching problem may have a different and complex surface of objective function, it is worth mentioning the ‘No Free Lunch (NFL) Theorem’ from Wolpert and Macready [9] which states that any pairs of different algorithms have identical average performance across all possible problems. This means that, at least from a theoretical perspective, it is impossible finding a general and efficient strategy for all history matching problems. Paradoxically, this fact is exactly what promotes the continuous interest in the area and makes valuable any work that proposes a different approach for solving history matching problems, contributing to overcome some of the challenges of the area.

This paper presents a dynamic decision-making optimization framework for history matching. The term ‘dynamic decision-making’ reflects the fact that, during the framework execution, the decision to generate a particular new solution is always guided and supported by the results of a continuous and dynamic analysis of the data from available solutions.

The proposed framework is different from previous approaches reported in the literature in the following aspects: it is not a stochastic method, since there is no randomness in its execution, nor it requires a large number of simulations to converge; it does not use a proxy model to substitute the flow simulator, so the results obtained with the framework are accurate at any moment of the execution; it is not a geostatistical process neither is primarily concerned with uncertainty reduction of the reservoir attributes. Rather, it is an optimization framework which follows a learning approach where the strategy is to dynamically analyze a set of observations (available solutions) to uncover input patterns (values of reservoir uncertain attributes) that lead to desired responses (good history matching for one or more wells) in the available solutions. The hypothesis of the proposed framework is that the input patterns identified during this ´learning-from-data’ process can be used while generating new solutions. The goal is not necessarily finding a set of perfect history-matched models, but verifying that the proposed dynamic decision-making optimization framework can, indeed, continuously learn from the data and, overtime, improve the history matching quality of an initial set of solutions. The choice for a ‘learning-from-data’ approach has been done with the purpose of giving the framework a potential to become an *specialist* on the structure of the history matching problem it is solving what, according to Ho and Pepyne in [10], would definitely improve the chances of it be considered superior when compared to other strategies for the same problem.

## 2 Dynamic decision-making optimization framework

**Fig 1:**
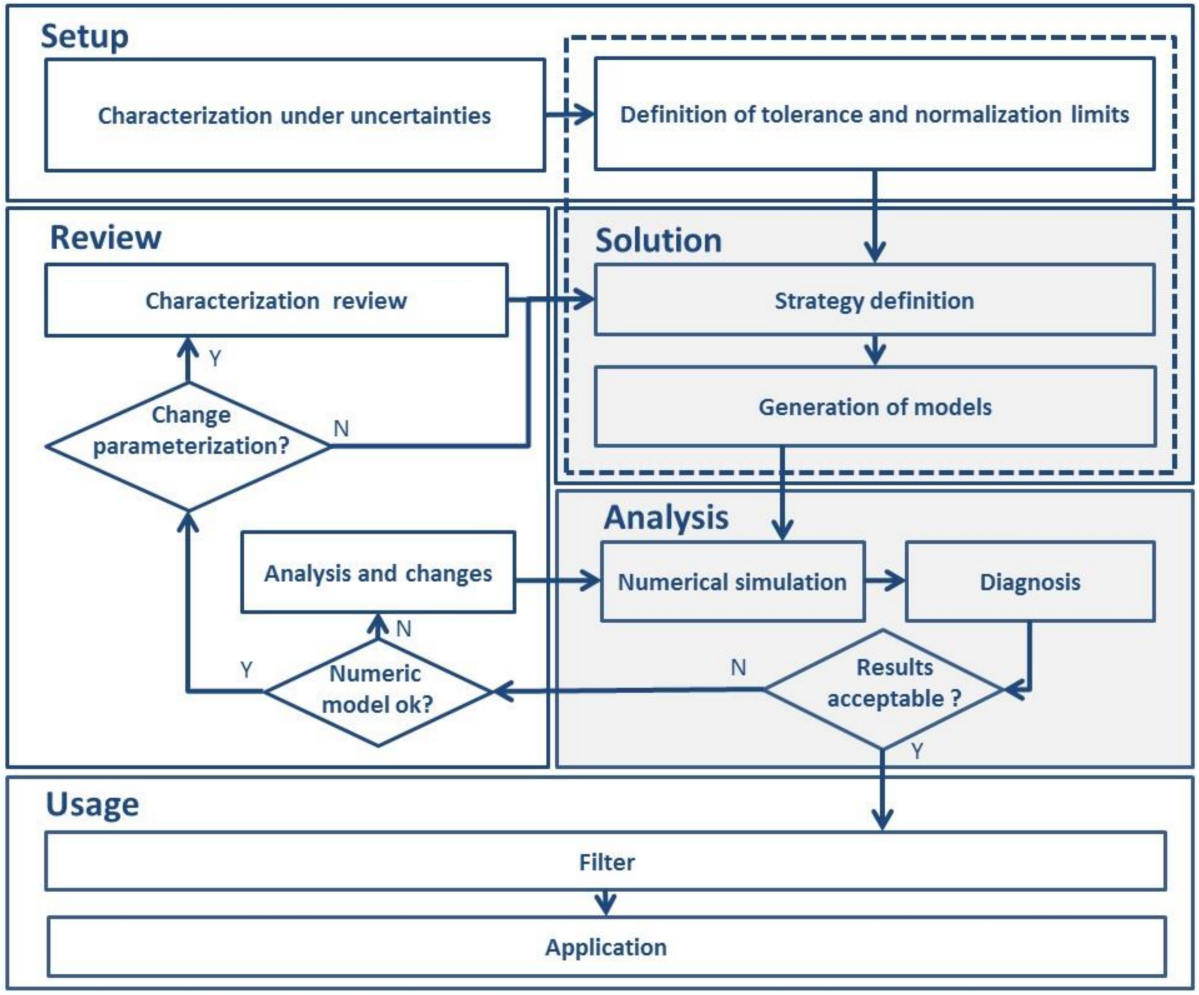
Generalized history matching methodology. Grey boxes highlight the components encompassed by the proposed dynamic decision-making optimization framework.

The dynamic decision-making optimization framework proposed in this work is designed in accordance with the generalized history matching methodology presented in Fig 1. This methodology is an evolution of the history-matching methodology introduced by Avansi and Schiozer [11], and intends to cover history matching algorithmic approaches (as is the case of the optimization framework proposed in this work) and approaches integrating history matching with uncertainty reduction. The steps introduced during the generalization are indicated by the dashed lines in Fig 1.

The proposed dynamic decision-making optimization framework encompasses the “Solution” and “Analysis” components of the generalized methodology. These are the components that have steps suitable to be automated and, hence, with potential for being improved by machine learning based solutions. The other components of the generalized methodology involve more specific knowledge from areas such as Reservoir Engineering and are not considered in this work.

**Fig 2:**
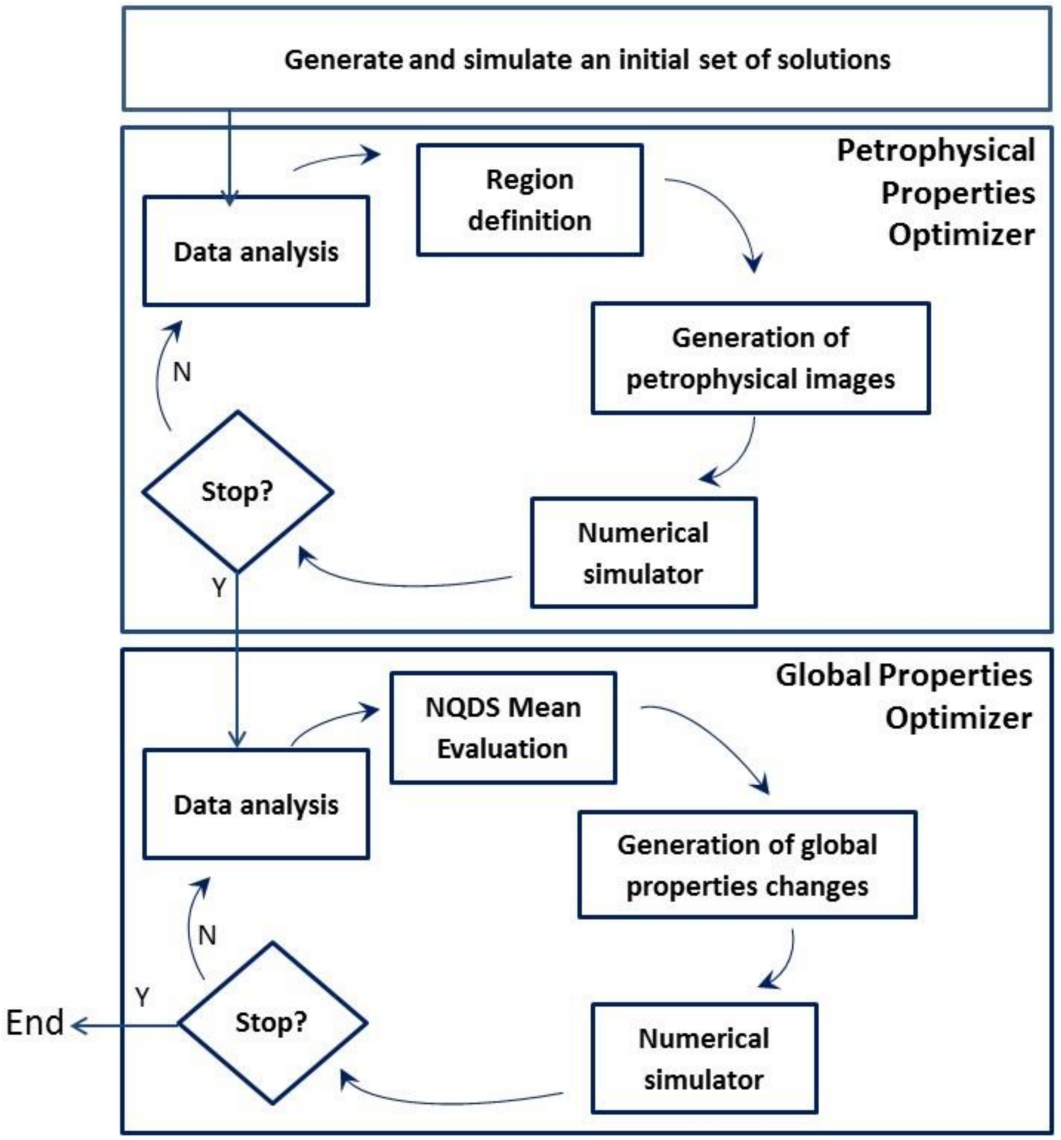
High-level workflow of the proposed optimization framework, including the two dynamic decision-making optimization components.

Fig 2 depicts the high-level workflow of the proposed dynamic decision-making optimization framework. The flow starts with the generation and simulation of an initial set of models (which can be of any size, provided it contains a good variability and models representative of the values of the uncertain attributes involved in the history matching). It then proceeds to a two-stage optimization approach, where each stage corresponds to a dynamic decision-making optimization component designed to handle different types of attributes. The first optimization stage, the Petrophysical Properties Optimizer, is focused on improving the models´ history matching quality through changes on petrophysical properties, which are defined for the reservoir grid blocks and may be changed locally in one or more reservoir regions. The second stage, the Global Optimizer, aims at optimizing the history matching objective function by changing the models´ global properties, which may impact the reservoir performance as whole.

Although Fig 2 shows the Petrophysical Optimizer being executed before the Global Optimizer, these two optimization stages are independent and the proposed framework is flexible regarding the execution order of its optimization components. Both Petrophysical Optimizer and Global Optimizer components work using the data of the current available models, so there is no restriction in the order they are executed. Of course, the solutions generated during the process (and the final results) may differ if the Petrophysical Optimizer is executed before the Global Optimizer, or vice-versa. The advantage of the proposed framework is that it is straightforward to intertwine the optimization stages.

From an algorithmic perspective, the proposed dynamic decision-making optimization framework follows a ‘learning-from-data’ paradigm which, according to Abu-Mostafa et al. in [12], is suitable for problems (such as the history matching problem) where there is no analytic solution, but there is a set of data that can be used to construct an empirical solution. Starting from an initial set of solutions, the proposed framework continuously analyzes the data of available solutions to identify patterns of input attributes that lead to good history matching responses. Once identified, these patterns are used in the generation of new models. Note the “Data analysis” step in Fig 2. It is present in both optimization stages and is the responsible for the dynamic analysis of the data from available solutions. This step is executed in each iteration of the framework optimizer components, and always precedes the step that generates the new models, so the results of the dynamic analysis can support the generation of the new models. The ultimate goal of the proposed framework is to generate better history-matched models based on the information (input patterns leading to good output response) that it can learn from the available solutions.

### 2.1 Generalized history matching methodology

The generalized history matching methodology introduced here (Fig 1) is based on the history matching methodology proposed by Avansi and Schiozer [11] and intends to provide a set of basic steps that can systematically be followed by anybody needing to solve a history matching problem. The goal is not defining a history matching solution method itself, but standardizing and outlining the steps one such method should consider.

The main concept of the generalized methodology is inherited from Avansi and Schiozer [11] and is the Normalized Quadratic Deviation with Sign (*NQDS*) quality indicator. The *NQDS* indicator is calculated for each output variable considered in the history matching process and for each model generated. It provides a measure of the misfit of the variable in the model and, more importantly, allows defining levels of matching quality, which are used to identify, and group, models with equivalent matching qualities for one or more variables.

The equations involved in the *NQDS* calculation are the following:

$${NQDS}_{x}=\frac{{QDS}_{x}}{{AQD}_{x}}$$ (1)

$${AQD}_{x}=\sum_{t=1}^{N_{times}}{\left({{Tol}_{x}*Hist}_{xt}+C_{x}\right)}^{2}$$ (2)

$${QDS}_{x}=\frac{{LD}_{x}}{\left|{LD}_{x}\right|}\sum_{t=1}^{N_{times}}{\left({Sim}_{xt}-{Hist}_{xt}\right)}^{2}$$ (3)

$${LD}_{x}=\sum_{t=1}^{N_{times}}\left({Sim}_{xt}-{Hist}_{xt}\right)$$ (4)

where:

*NQDS*_*x*_ = normalized quadratic deviation with sign of variable *x*, representing the misfit component associated with this variable;
*QDS*_*x*_ = quadratic deviation with sign of variable *x*;
*AQD*_*x*_ = acceptable quadratic deviation of variable *x*, used to normalize the *QDS*_*x*_;
*Tol*_*x*_ = acceptable tolerance, in [0,1], for variable *x* be considered good with respect to historical data;
*Hist*_*xt*_ = historical data of variable *x* in time *t*;
*C*_*x*_ = constant added to prevent *AQD*_*x*_ of being too small or restrictive in the cases where the data of a variable *x* is zero during a long time interval;
*LD*_*x*_ = linear deviation of variable *x*;
*Sim*_*xt*_ = simulated data of variable *x* in time *t*.

**Fig 3:**
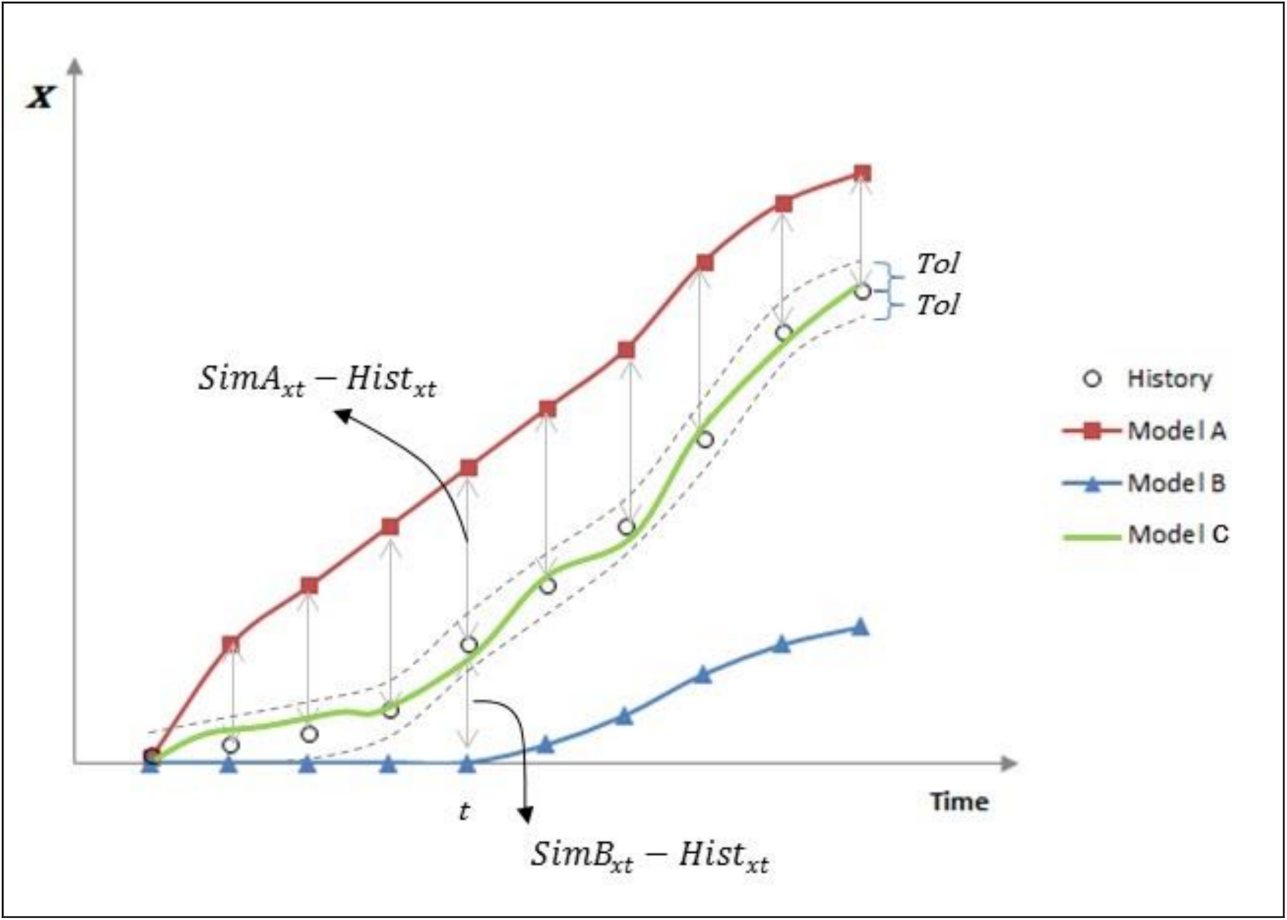
Graphical example of the basic concepts involved in the NQDS calculation.

Fig 3 depicts graphically some of the basic concepts involved in the *NQDS* calculation. For each time t, the difference (*Sim*_*xt*_ − *Hist*_*xt*_) represents the linear distance between the simulation data and the history data. The linear deviation *LD*_*x*_ is the sum, overtime, of each of these differences. Curves which are mainly above the history data, such as the one corresponding to Model A in Fig 3, have a positive value of *QDS*_*x*_ and hence of the *NQDS*_*x*_ indicator. Similarly, curves mainly below the history data, such as the one from Model B, have negative values of *QDS*_*x*_ and *NQDS*_*x*_. A perfect matching for a variable *x* has *LD*_*x*_ 0. Since this is very difficult to achieve, especially when the history matching process calibrates many variables at the same time, the *Tol*_*x*_ defines an acceptable range (dashed lines in Fig 3) within which the matching quality, although not perfect, is considered excellent. *NQDS*_*x*_ values in [-1,+1] interval indicate curves, such as the one from Model C in Fig 3, which are mainly or totally contained in the *Tol*_*x*_ defined acceptable range. As a general rule, for any simulation model *Sim*, the bigger (smaller) the positive (negative) value of *NQDS*_*x*_, the further from the acceptable range is the curve of *x* in *Sim*. The acceptable range is defined for each variable *x* involved in the history matching process and may vary according to the confidence in the variable history data, the variable importance, or the desired solution quality.

The generalized history matching methodology defines six quality levels (Table 1.) for the *NQDS*_*x*_ indicators. These levels reflect the quality of the matching, and have been defined based on the authors experience with history matching problems. The ultimate goal of the methodology is to find a set of solutions in which the *NQDS*_*x*_ values are in the “Excellent” level for all variables *x* considered in the history matching process.

**Table 1:** Quality levels for *NQDS*_*x*_.

| *NQDS*_*x*_ |	in [0,1]	in] 1,2]	in] 2,3]	in] 3,5]	in] 5,10]	not in
						[-10,10]
Quality level	Excellent	Very good	Good	Regular	Bad	Very bad

The following subsections briefly describe the five main components of the generalized history matching methodology (Fig 1) and their encompassed steps. A detailed description about the original methodology, on which the one presented here was based, can be found in [11].

#### 2.1.1 Setup

The “Setup” component is responsible for the initial steps in the history matching process:

- **Characterization under uncertainties**
  Step responsible for the definition of a reservoir model and the uncertain attributes that will be considered during the history matching process. The corresponding range, levels and probability distribution function of each uncertain attribute are also defined at this moment.
- **Definition of tolerance and normalization limits**
  Step in which the *Tol*_*x*_ and *C*_*x*_ values used in Eq (2) are defined to delimit the acceptable range for each variable *x*. Once defined, these values are kept constant during all the history matching process.

#### 2.1.2 Solution

The “Solution” component is the core of the history matching process and comprises the following steps:

- **Strategy definition**
  Step which involves choosing the strategy for the history matching process. There are many possibilities to find better adjusted models. Sampling techniques based on the probability distribution function of the uncertain attributes, optimization methods whereby new models are generated with the goal of minimizing a history matching objective function, and strategies focused on the reduction of the attributes uncertainties are some of the most common approaches adopted.
- **Generation of models**
  This is the step where new simulation models are generated according to the strategy defined in the previous step. In case of uncertainty reduction, for example, the new models can be generated combining the values of the uncertain attributes through a sampling algorithm such as the Discretized Latin Hypercube with Geostatistical Realizations (DLHG) method from Schiozer et al. [13]. In the case of an optimization strategy, the models are generated following the optimization goals. The dynamic decision-making optimization framework proposed in this work generates better calibrated models using the patterns of attributes present in good quality solutions identified among all available models.

#### 2.1.3 Analysis

The main purpose of the “Analysis” component is to verify the quality of the models generated by the “Solution” component. The steps of this component are:

- **Numerical simulation**
  Step in which the new models are simulated.
- **Diagnosis**
  Step responsible for the calculation of the *NQDS*_*x*_ quality indicators for all the simulated models.
- **Analysis of results**
  Verification step in which the quality indicators previously calculated are analyzed. If there are models with *NQDS*_*x*_ indicators in acceptable levels for all variables *x*, the flow proceeds to the “Usage” component; otherwise, it goes to the “Review” component.

#### 2.1.4 Review

The “Review” component attempts to identify possible causes for the current available models still not being considered good enough in terms of history matching quality. The steps of this component are:

- **Numeric model verification**
  Step that checks the consistence of the numeric aspects used in the simulation models. Verifying the numeric aspects involve finding possible inconsistences in well dynamic data (history), well completion positions, among others, and if a problem is identified, the flow proceeds to the “Analysis and changes” step.
- **Analysis and changes**
  Step in which the numeric model is changed to fix consistence problems identified in the previous step.
- **Analysis of parameterization**
  When the numeric model is correct, unacceptable results in the “Analysis” component (Section 2.1.3) may be related to problems in the initial model characterization. This step checks if there is any problem with the current parameterization which may be preventing finding good history matched solutions.
- **Characterization review**
  This step fixes parameterization problems identified in the previous step. The model characterization is reviewed and a new uncertain attribute may be inserted in the model, or the variation range and/or the levels of the existent attributes may be modified.

#### 2.1.5 Usage

The “Usage” component is the final component of the generalized history matching methodology (Fig 1). At this stage, the best history matched models are selected and used to predict future reservoir performance, supporting the decision-making process on other reservoir engineering tasks. The steps of this component are:

- **Filter**
  Step that selects the models for which the *NQDS*_*x*_ quality indicators, for all variables *x*, are in an acceptable (ideally “Excellent”) level.
- **Application**
  The models selected in the previous step are used to predict the real reservoir performance and to support decisions on tasks such as economic analysis and production strategy.

The dynamic decision-making optimization framework proposed in this work (Fig 2) covers the steps in the “Solution” and “Analysis” components of the generalized history matching methodology described in this section. The optimization strategy adopted involves the minimization of an objective function which is detailed in Section 2.2. The new models are generated using attributes patterns extracted from good-quality solutions identified during the “Data Analysis” step described in Section 2.3. The optimization is carried out by the two optimization components, described, respectively, in Section 2.4 (Petrophysical Properties Optimizer) and in Section 2.5 (Global Properties Optimizer).

### 2.2 Objective function

Optimization approaches, such as the optimization framework proposed in this work, need a formal way to evaluate the quality of the solutions found during the optimization process.

The generalized history matching methodology described in Section 2.1 prescribes that an acceptable solution is one where the *NQDS*_*x*_ indicators are in an acceptable level for all variables *x* involved in the history matching process. From an optimization perspective, each variable to be matched may be seen as a different objective-function, and these multiple functions can be optimized simultaneously by a multi-objective optimization approach. The framework proposed in this work, however, is a single-objective optimization strategy where the multiple variables to be matched are considered together during the optimization process and the quality of a given solution (model) is measured through a single misfit value which expresses the proximity of the simulated data when compared with the historical data: the lower the misfit value the better the solution. The goal of the single-objective history matching optimization process is finding solutions that minimize the misfit value, also referenced as the history matching objective function.

In this work, the misfit value M to be minimized by the optimization framework is defined as the Euclidean norm of the vector containing the misfit components of each well variable (well rates and pressure) and is calculated using the *NQDS*_*x*_ indicators (Section 2.1) through the following equation:

$$M=\sqrt{\sum_{x=1}^{N_{var}}{\left({NQDS}_{x}\right)}^{2}}$$ (5)

where:

*M* = misfit value to be minimized;
*N*_*var*_ = total number of well variables (number of variable per well multiplied by the number of wells) considered in the history matching procedure;

### 2.3 Data Analysis step

The “Data Analysis” step is a core step in the proposed dynamic decision-making optimization framework (Fig 2) and it is present in both the *Petrophysical Properties Optimizer* and the *Global Properties Optimizer* components. It has two responsibilities: (i) to evaluate the history matching quality of the available models, calculating, for each of them, the misfit value defined in Section 2.2; (ii) to identify among the available models, attribute patterns present in good-quality solutions. In order to achieve this objective, once the misfit value has been calculated for each model, the models are sorted using different criteria so that, in the “Data Analysis” step output, the following types of solutions can be identified:

- the best available model: the one with the lowest misfit value;
- the best model for each particular well: the one with the lowest Euclidean norm of the vector containing only the misfit component values (*NQDS*_*x*_ indicators) associated with the variables of the given well;
- the best model regarding each particular series (well rate or pressure): the one with the lowest Euclidean norm of the vector containing only the misfit component values (*NQDS*_*x*_ indicators) associated with the variables of the given series.

The above three types of “best” solutions and the patterns of attributes they contain are used to support the decision-making of the optimizer components when they generate new simulation models.

### 2.4 Petrophysical properties optimizer

The petrophysical properties optimizer component is the first optimizer component of the proposed dynamic decision-making framework (Fig 2). It aims at finding better history matched models focusing on changes on the values of uncertain attributes associated with petrophysical properties.

In a reservoir model, petrophysical properties such as porosity and horizontal and vertical permeabilities are usually correlated. For this reason, they are frequently defined together in what is called petrophysical realization or petrophysical image: a set of values defining the petrophysical properties for each reservoir grid block.

The petrophysical properties optimizer proposed in this work uses the data from the petrophysical realizations associated with the best global model and the best model for each well, identified during the Data Analysis step, to generate new petrophysical realizations and the corresponding models associated with each one of them.

The process of generating the new petrophysical images uses unsupervised learning to partition the reservoir grid into a set of disjoint regions (grid segments), each of which containing one and exactly one well. This partitioning is done by the “Region Definition” step (Section 2.4.1) and it is a critical part of the petrophysical properties optimizer component. It depends on the petrophysical realization of the best available solution (Section 2.3) and, since it is called at each optimizer iteration, as far as the petrophysical realization of the best solution changes from one iteration to another, the set of reservoir regions also changes dynamically from one iteration to another.

The new petrophysical images are generated using the petrophysical realization of the best available solution (Section 2.3) and replacing the data of its regions, one region at a time or all at once, by the corresponding region data extracted from the petrophysical realization of the best available solution for the particular well contained in the region. In this way, in each iteration of the petrophysical optimizer, the number of new images generated is potentially equal to the number of wells plus one.

The new models, using the generated petrophysical images, are simulated and the process continues until a stop criterion (max number of iterations or no new image generated) is reached.

It is worth mentioning here that, although geological realism has not been a primary concern of the proposed optimization framework, the way the new petrophysical images are generated by the Petrophysical Optimizer is based on two aspects that aim at adhering to the geological consistence of the models. The first aspect is that the images are generated using patches collected from geological consistent realizations generated previously (the ones from the best global solution and from the best available solution for each well). The second aspect is that the shape of the patches is the shape of the regions defined by the ‘Regions Definition’ step (Section 2.4.1), which intentionally groups reservoir grid blocks spatially close to each other and with similar values of petrophysical properties, maintaining the consistence of spatial correlation.

#### 2.4.1 Regions Definition step

The “Regions Definition” step is a clustering algorithm based on the spatial coordinates and petrophysical properties of the reservoir model grid blocks. Its goal is to partition the reservoir grid into a set of disjoint regions, such that each region contains exactly one well, and the union of the regions corresponds to the entire reservoir.

Dividing the reservoir into regions is a common task during history matching. One frequently used approach is to define the regions using Voronoi polygons around the wells (Caeiro et al., 2015). In this case, each reservoir grid block is assigned to the region containing the well which is closest to the block. The distance calculation involves only the well and the block coordinates.

This work proposes a different approach for partitioning the reservoir into regions. Instead of using only the wells and blocks coordinates to decide if a block shall belong to a particular region, the idea here is to also consider the values of the petrophysical properties of the grid blocks. The resulting region containing a particular well will not only be formed by grid blocks near to the well, but will also contain the blocks whose petrophysical properties are closest to the properties of the well block.

The proposed partitioning strategy can be done using petrophysical values extracted from any available petrophysical realization. Nevertheless, in this work, the petrophysical values used in the “Regions Definition” step are always the ones present in the best available solution.

The algorithm of the *Regions Definition* step works on a space ***S*** in R^n^, containing the set of vectors ***v*** = (*v*_*1*_, *v*_*2*_, …, *v*_*n*_) constructed as follow:

1. There is a vector ***v*** for each reservoir grid block;
2. *v*_*1*_, *v*_*2*_, *v*_*3*_ are the *x*, *y*, *z* coordinates of the grid block;
3. *v*_*4*_, *…*, *v*_*n*_ are the normalized values of the petrophysical properties of the grid block.

**Fig 4:**
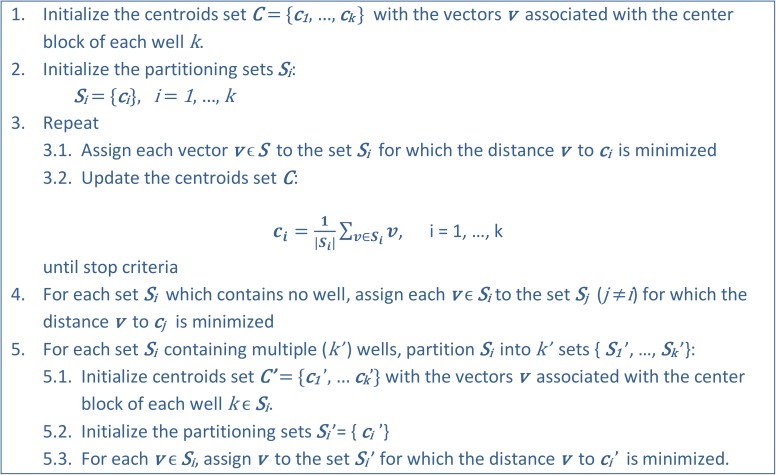
The Regions Definition algorithm.

The pseudo-code of the proposed algorithm is presented in Fig 4. Steps 1 to 3 are essentially the traditional k-means algorithm [14] and partitions the space S into k (number of wells) sets, S = {S1, S2, …, Sk}, such that the sum of the intra-set distances (Eq 6) is minimized.

$$\sum_{i=1}^{k}\sum_{v\in S_{i}}\sqrt{{\left(v_{1}-c_{i1}\right)}^{2}+\ldots +{\left(v_{n}-c_{in}\right)}^{2}}$$ (6)

Steps 4 and 5 are post-processing steps to ensure that each set in the final partition contains one and exactly one well.

**Fig 5:**
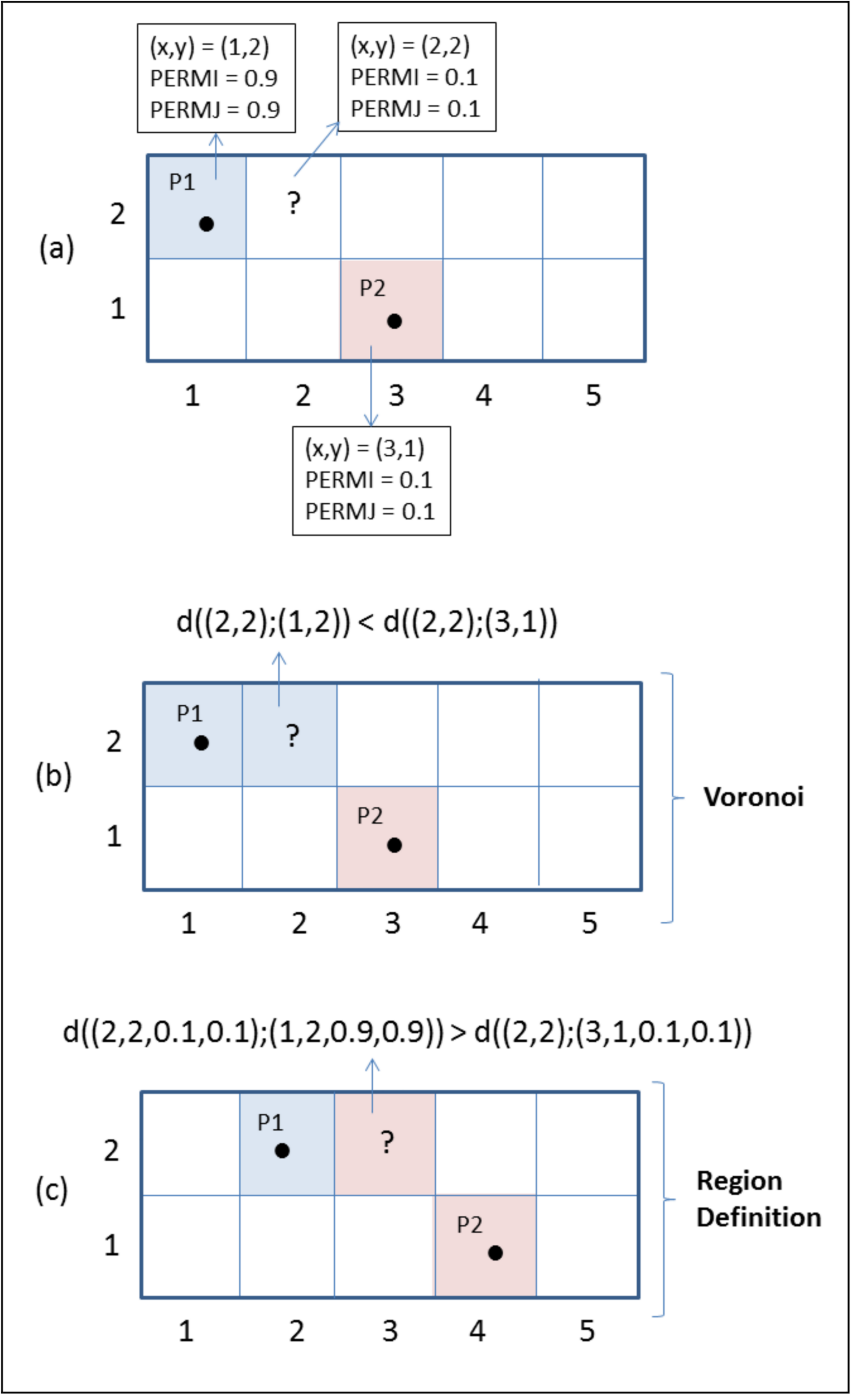
Illustration of differences between Voronoi and Region Definition strategies for partitioning the reservoir grid into regions. (a) moment when block “?” needs to be assigned to the region containing P1 well or to the region containing P2 well; (b) assignment of “?” block to the region containing P1 well using Voronoi strategy; (c) assignment of “?” block to the region containing P2 well using the Region Definition strategy.

Fig 5 illustrates the difference between the traditional Voronoi strategy and the Region Definition strategy proposed in this work for partitioning the reservoir into regions. The example shows how a particular grid block is indeed assigned to different regions depending on the strategy used. Fig 5(A) shows a reservoir with two wells, P1 and P2, and ten grid blocks, and highlights the moment when one needs to decide if the “?” block shall be assigned to the region of P1 well or to the region of P2 well. For simplicity, the example uses only the x,y coordinates of the grid blocks and two petrophysical properties (PERMI and PERMJ). Fig 5(B) shows that, using the Voronoi strategy that considers the distances calculated using only the block coordinates, the “?” block is assigned to the region of P1 well. On the other hand, Fig 5(C) shows that, using the Region Definition strategy proposed in this work, that considers the distances calculated using the block coordinates and petrophysical properties similarity, the “?” grid block is assigned to the region of P2 well.

### 2.5 Global properties optimizer

The global properties optimizer component is responsible for iteratively changing the values of the uncertain global attributes of the best available solution (Section 2.3), with the purpose of improving its history matching quality.

The process of changing the global attribute values follows an expectation-maximization approach where, at every iteration, the promising levels of the attributes are evaluated (expectation) and, eventually, the best global solution is changed based on the gathered information (maximization).

The identification of the global attributes promising levels is based on the analysis of the impact these levels may have on the misfit components of the history matching solution. This analysis is dynamically performed in each iteration by the “NQDS Mean Evaluation” step and it is the core of the dynamic decision making of the proposed optimizer component.

Let *y*_*ij*_ represent the *j*^*th*^ level of an uncertain global attribute *y*_*i*_. For each well variable *x*, and global attribute level *y*_*ij*_, the “NQDS Mean Evaluation” step calculates the ${\bar{NQDS}}_{xy_{ij}}$ value as the mean of the misfit components *NQDS*_*x*_ (Section 2) considering all models that have the attribute *y*_*i*_ in level *j*.

The ${\bar{NQDS}}_{xy_{ij}}$ value can be understood as an indicator of the average contribution to the objective function of the misfit component associated with well variable *x*, in models which have attribute *y*_*i*_ in level *j*. High absolute values of ${\bar{NQDS}}_{xy_{ij}}$ may indicate that, on average, having attribute *y*_*i*_ in level *j* leads to a high misfit of well variable *x* in a solution.

The proposed global optimizer component uses the ${\bar{NQDS}}_{xy_{ij}}$ values to decide if a change in the value of a global attribute is promising to generate better quality solutions. In each iteration, the new models are generated using the following strategy:

1. Let *y*_*il*_ represent the level of the uncertain global attribute *y*_*i*_ in the best available solution (Section 2.3), and let *y*_*i(l-1)*_ and *y*_*i(l+1)*_ be, respectively, the previous and next levels of *y*_*il*_.
2. If $\left|{\bar{NQDS}}_{xy_{i(l-1)}}\right|<\left|{NQDS}_{xy_{il}}\right|$, for at least one well variable *x*, generate a new model changing the value of attribute *y*_*i*_ to its value on level *y*_*i(l-1)*_;
3. If $\left|{\bar{NQDS}}_{xy_{i(l+1)}}\right|<\left|{NQDS}_{xy_{il}}\right|$, for at least one well variable *x*, generate a new model changing the value of attribute *y*_*i*_ to its value on level *y*_*i(l+1)*_.

In order to illustrate the strategy of the Global Optimizer, consider the situation where it is being executed with a scenario that has only two global uncertain attributes (A and B), which have been discretized into 3 and 5 levels, respectively, as shown in Table 2 and Table 3 below.

**Table 2:** Discrete levels of global uncertain attribute A.

Level	0	1	2
Value	1.0	3.0	5.0

**Table 3:** Discrete levels of global uncertain attribute B.

Level	0	1	2	3	4
Value	3170	3172	3174	3176	3178

Consider now that in the current best solution, the levels of attributes A and B are: A 1 and B 3. The Global Optimizer will attempt to generate new models changing the levels of A and B to their neighbor levels. The potential new models are: A 0 B 3, A 2 B 3, A 1 B 2 and A 1 B 4. Note that only one global attribute is changed to generate a new model. The intelligence of the Global Optimizer is that these potential new models will only effectively be generated *if and only if* the neighbor level, on average, improves the *NQDS*_*x*_ indicator of at least one of the variables involved in the process. For the particular potential new models of this example, A 0 B 3 will only be generated if, considering all the available models having A in level 0, the average of the indicator *NQDS*_*x*_ is better than the corresponding value in the current best solution, for at least one variable *x*. Similarly, the model A 2 B 3 will only be generated if, considering all available models having A in level 2, the average of the *NQDS*_*x*_ indicator is better than the corresponding value in the current best solution, for at least one variable *x*. The same reasoning applies to models A 1 B 2 and A 1 B 4.

The number of new models generated by the global optimizer in each iteration can be twice the number of global attributes present in the best available solution. Nevertheless, the reasoning behind the presented strategy is that a new model is generated if, and only if, the change on the uncertain global attribute value, on average, intends to improve the misfit component associated with at least one of the well variables. This allows, on one side, setting the focus of the global optimizer component on the generation of promising solutions, and on the other side, potentially reducing the number of new simulations, as non-promising changes are not considered. It is also worth mentioning that, since the analysis to identify promising levels is performed dynamically, in each iteration, and considers the current available models, the fact that a level of an uncertain attribute is rejected at one moment does not mean that it cannot be reconsidered and accepted in a further moment.

Finally, in each iteration of the Global Properties Optimizer, the new generated models are simulated and the process continues until a stop criterion (max number of iterations or local minimum found) is reached.

## 3 Experiments and results

This section describes the experiments carried out to validate the use of the proposed dynamic decision-making optimization framework to improve the quality of history matching solutions.

The experiments were organized in three rounds:

- **Preliminary experiments**, with the purpose of configuring the execution order of the framework optimizer components, and performing basic validation on the regions delimited by the “RegionDefinition” strategy (Section 2.4.1), and on the petrophysical images generated by the Petrophysical Optimizer (Section 2.4).
- **Experiments with the complete optimization framework**, with the purpose of showing the performance of the implemented framework on datasets from a benchmark reservoir model, and comparing the results obtained with the framework against the ones from a recent literature history matching approach on the same benchmark.
- **Experiments with the petrophysical properties optimizer**, focused on comparing the improvements on history matching solutions obtained with the proposed “Region Definition” strategy (Section 2.4.1) for partitioning the reservoir into regions, versus the improvements obtained when using the traditional Voronoi partitioning approach.

The dynamic decision-making optimization framework was implemented in JAVA (1.7.0_40) and coupled to MERO program which is being developed by UNISIM group (http://www.unisim.cepetro.unicamp.br/). MERO is a set of tools that enables the creation of workflows to support the development and management of petroleum reservoirs. The provided tools aim to automate tasks that use numerical reservoir simulation and are common process in areas such as history matching, production strategy and uncertainty and risk analysis. MERO supports many simulators and in this work was configured to use IMEX simulator (2012.10) from Computer Modelling Group (http://www.cmgl.ca/).

All the experiments were conducted using datasets based on the UNISIM-I-H benchmark case.

### 3.1 UNISIM-I-H benchmark

UNISIM-I-H is a benchmark case for history matching. It is described in [11] and is a synthetic model based on real data from Namorado Field, located in Campos Basin, Brazil.

**Fig 6:**
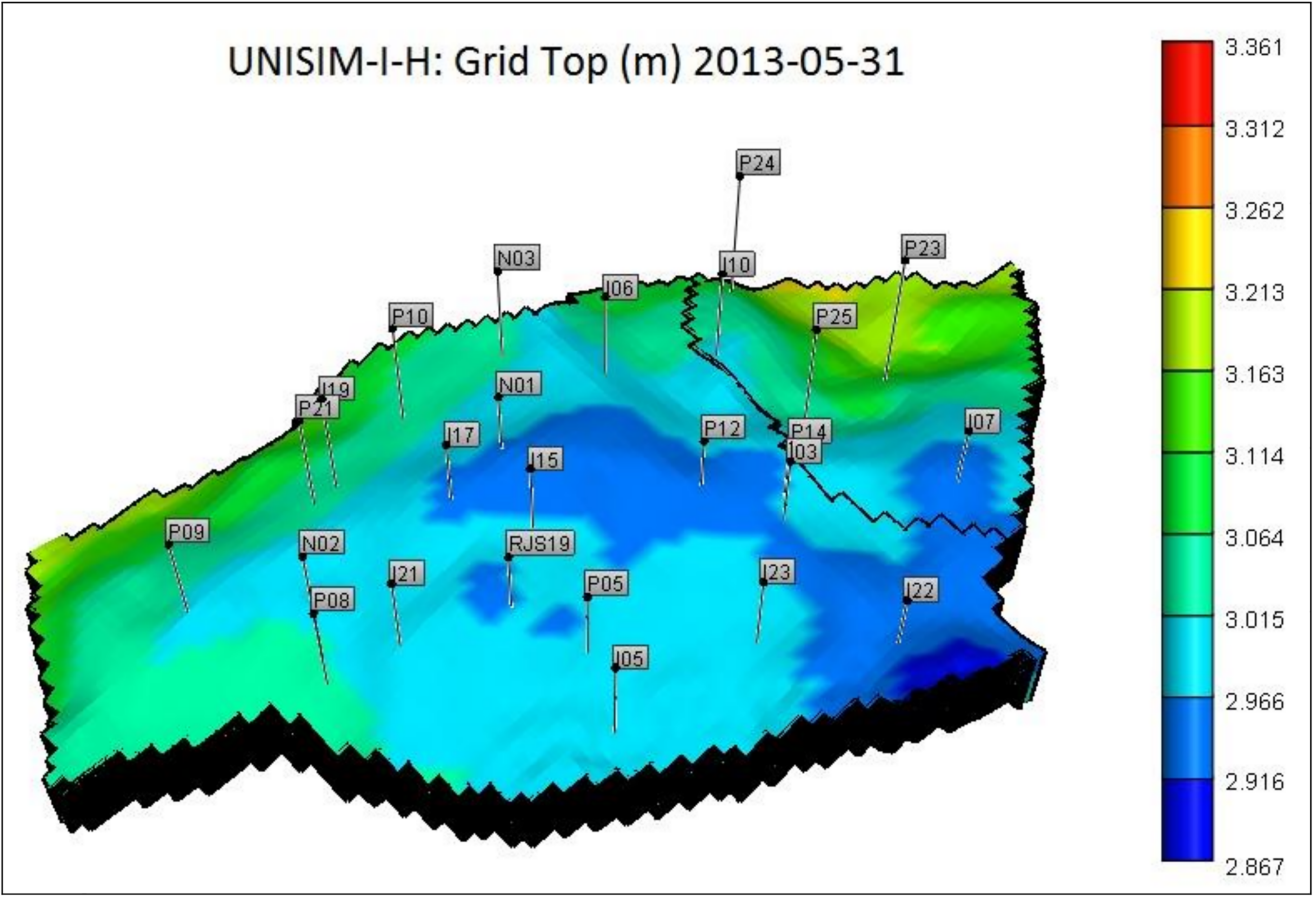
UNISIM-I-H well distribution in reservoir grid top.

The model has 25 wells, 14 producers and 11 injectors. Fig 6 shows the well distribution in the reservoir grid top.

The reservoir has two regions, isolated from each other through a sealing fault (black line in Fig 6). Region 1 is the one located in the west of the sealing fault, and region 2 is the smallest one located in the east.

The model grid has 81 x 28 x 50 blocks, each measuring 100 x 100 x 8 m.

The uncertain properties of UNISIM-I-H model are summarized in Table 4. The “# Levels” column shows the number of levels considered for each property in this work. For the first three properties, the uncertain levels come directly from the UNISIM-I-H model characterization (http://www.unisim.cepetro.unicamp.br/benchmarks/br/unisim-i/unisim-i-h): there is one file for each uncertain level of these properties For the last three properties, which are continuous properties, the levels are the result of a discretization, made from the property probability distribution functions defined in Table 5.

**Table 4:** Uncertain properties considered in the history matching of UNISIM-I-H model.

Nomenclature	Description	# Levels
*Petro*	Petrophysical properties (porosity, horizontal and vertical permeabilities, and net to gross ratio)	500
*K*_*rw*_	Water relative permeability	5
${PVT}_{R_{2}}$	Region 2 PVT data	3
${WOC}_{R_{2}}$	Region 2 oil-water contact	5
*CPOR*	Rock compressibility	5
*Kz_c*	Vertical permeability multiplier	5

**Table 5:** Uncertainty ranges of continuous properties.

Property (unity)	PDF
${WOC}_{R_{2}}$ (m)	0, *x* < 3169
	$\frac{x-3169}{25}$, 3169 ≤ *x* < 3174
	$\frac{3179-x}{25}$, 3174 ≤ *x* ≤ 3179
	0, *x* > 3179
*CPOR* * 10^−6^(cm2/kgf)	0, *x* < 10
	$\frac{x-10}{1849}$, 10 ≤ *x* < 53
	$\frac{96-x}{1849}$, 53 ≤ *x* ≤ 96
	0, *x* > 96
*Kz_c* (-)	0, *x* < 0
	$\frac{2x}{4.5}$, 0 ≤ *x* < 1.5
	$\frac{6-2x}{4.5}$, 1.5 ≤ *x* ≤ 3
	0, *x* > 3

The *Petro* properties are the ones manipulated by the Petrophysical Optimizer. The other 5 properties (*K*_*rw*_, ${PVT}_{R_{2}}$, ${WOC}_{R_{2}}$, *CPOR*, *Kz_c*) are the global attributes considered during the Global Optimizer execution.

The available history data covers a period of 11 years of reservoir production and the well variables to be matched during the history matching procedure are:

- oil production rate (QO), water production rate (QW) and gas production rate (QG) for producer wells;
- water injection rate (QWI) for injector wells;
- bottom hole pressure (BHP) for all wells.

Considering that the UNISIM-I-H model has 14 producer wells and 11 injector wells, this leads to a total of 78 misfit variables in the objective function (Eq (5), Section 2.2) to be minimized during the history matching process.

In the generalized history matching methodology (Section 2.1), the acceptable range within which the matching quality is considered “Excellent” can be defined individually for each variable *x*, through the use of different values for the *Tol*_*x*_ and *C*_*x*_ constants (Eq (2), Section 2.1). In this work, however, the acceptable ranges were considered the same for all variables of a particular series (e.g., the acceptable range for the oil rate (QO) variables for all producers was defined using *Tol* = 0.1 and *C* = 0). Table 6 summarizes the *Tol* and *C* values adopted for each series during the experiments.

**Table 6:** Tol and C values (by series) used to calculate the acceptable quadratic deviations in Eq (2).

	Series				
	QO	QW	QG	QWI	BHP
*Tol*	10%	10%	10%	5%	10%
*C*	0	20	0	0	0

### 3.2 Datasets

With the purpose of grouping the scenarios used in the validation of the proposed framework and facilitating the presentation of the experimental results, two datasets (UNISIM-I-H-A and UNISIM-I-H-B) were created based on the UNISIM-I-H benchmark case.

The datasets are similar in the sense that each one has five scenarios and each scenario initially comprises a set of 100 solutions for the history matching problem of UNISIM-I-H case.

The datasets will be public upon publication of this paper, what may facilitate the comparison between the results presented in this paper with the ones obtained with different history matching strategies in future works.

#### 3.2.1 UNISIM-I-H-A

This dataset contains five scenarios, each of which containing 100 models. The models in each scenario were generated using the Discretized Latin Hypercube with Geostatistics (DLHG) method [13] to sample the uncertainties described in Table 4. The number of distinct petrophysical images in each scenario was set to 100 (smaller than the 500 originally defined in Table 4) based on the work of Schiozer et al. [13], which mentions that 100 to 200 petrophysical realizations are often sufficient to represent the petrophysical properties’ uncertainties in a risk analysis process applied to the UNISIM-I-H model.

Table 7 presents the UNISIM-I-H-A scenarios with their corresponding petrophysical properties levels. The levels of the non-petrophysical properties and their discrete probabilities are the same for all scenarios and are summarized in Table 8.

**Table 7:** UNISIM-I-H-A scenarios and their levels of petrophysical properties.

Scenario	Petrophysical Properties Levels (100 equiprobable images)
S1	im0 … im99
S2	im100 … im199
S3	im200 … im299
S4	im300 … im399
S5	im400 … im499

**Table 8:** Levels and discrete probabilities for non-petrophysical properties.

Property	Levels				
	(Probability)				
*K*_*rw*_	krw0	krw1	krw2	krw3	krw4
	(0.20)	(0.20)	(0.20)	(0.20)	(0.20)
${PVT}_{R_{2}}$	pvt0	pvt0	pvt1	-	-
	(0.33)	(0.34)	(0.33)	-	-
${WOC}_{R_{2}}$	woc0	woc1	woc2	woc3	woc4
	(0.10)	(0.20)	(0.40)	(0.20)	(0.10)
*CPOR*	cpor0	cpor1	cpor2	cpor3	cpor4
	(0.10)	(0.20)	(0.40)	(0.20)	(0.10)
*Kz_c*	kzc0	kzc1	kzc2	kzc3	kzc4
	(0.10)	(0.20)	(0.40)	(0.20)	(0.10)

#### 3.2.2 UNISIM-I-H-B

The UNISIM-I-H-B dataset is similar to the UNISIM-I-H-A dataset. It also contains five scenarios, S6 to S10, each of which containing 100 models generated in the same way used for the models in UNISIM-I-H-A: Discretized Latin Hypercube with Geostatistics (DLHG) method [13] to sample the uncertainties, non-petrophysical properties as defined in Table 8 and 100 distinct petrophysical images in each scenario. The difference here is that, instead of having the images in each scenario pre-defined as was the case in UNISIM-I-H-A (Table 7), in the scenarios of UNISIM-I-H-B, the 100 images of each scenario were randomly selected from the 500 ones available from the UNISIM-I-H model characterization.

### 3.3 Evaluation metrics

In order to evaluate the performance of the dynamic decision-making framework, the following two metrics were considered:

- Misfit value (Eq (5), Section 2.2) of the best available solution
- Number of models by max |*NQDS*_*x*_| for the 100 best models

For each scenario, the metrics were measured in three different points:

1. after the simulation of the initial set of models, just before the execution of the petrophysical properties optimizer and global properties optimizer components;
2. after the execution of the petrophysical properties optimizer component;
3. after the execution of the global properties optimizer component.

### 3.4 Preliminary experiments

**Fig 7:**
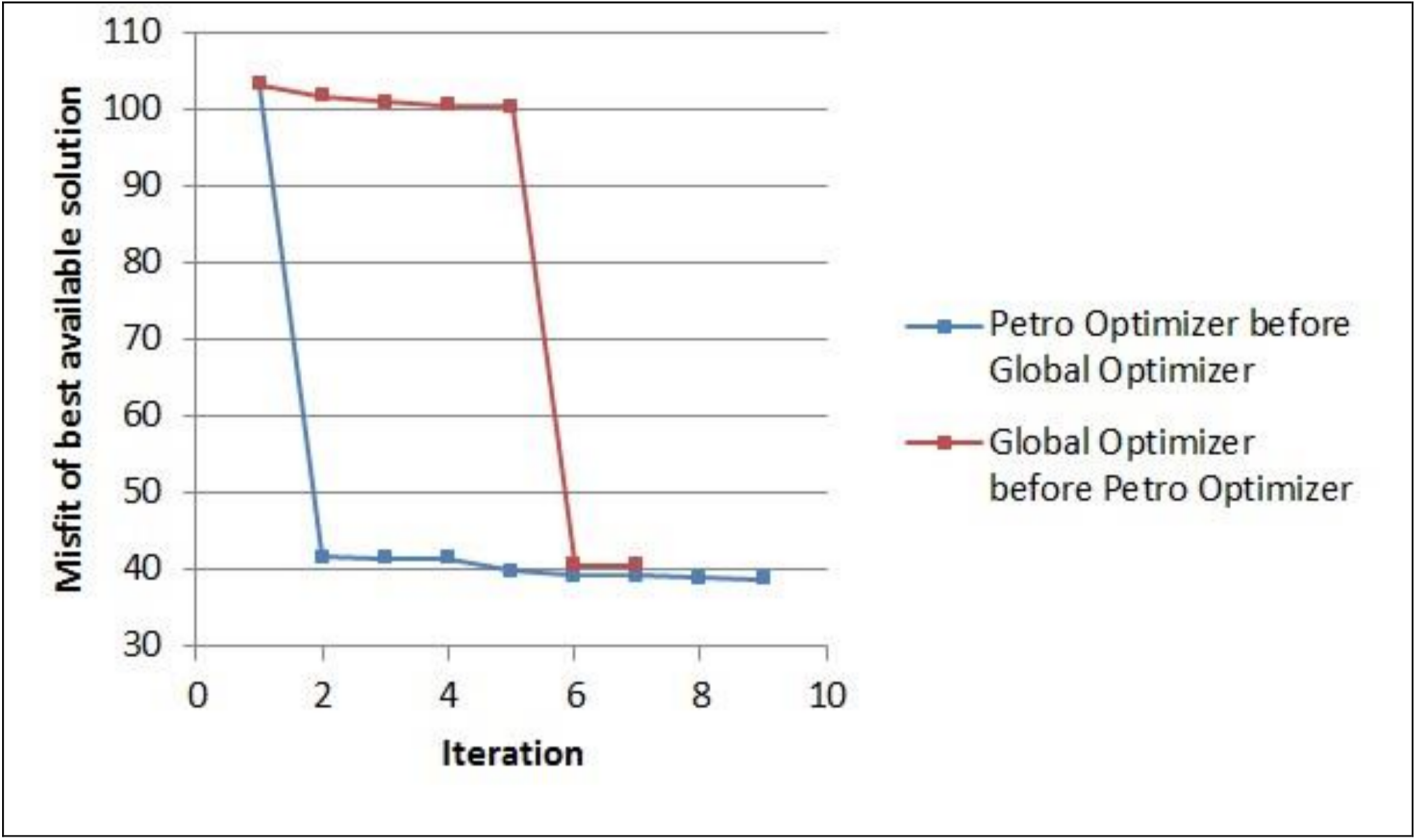
Convergence plots of the misfit of best available solution in UNISIM-I-H-A S1 scenario.

In the first experiment, the optimization framework was executed with two possible configurations of its optimizer components: Global Optimizer before Petrophysical Optimizer, and Petrophysical Optimizer before Global Optimizer. Fig 7 shows the convergence plots of the misfit of the best available solution in the UNISIM-I-H-A S1 scenario in the two configurations. The plots for the other scenarios are similar to this one presented here.

The first important thing to note in Fig 7 is that the steepest decrease in the misfit value, in both configurations, was obtained during the execution of the Petrophysical Optimizer: iteration 1, when the Petro Optimizer was executed before the Global Optimizer; and iteration 5 when the Global Optimizer was executed first. Also, despite the final misfit of the best available solution obtained with each configuration being very similar, in this particular experiment, executing the Petrophysical Optimizer first (blue curve) led to a slightly better result. For this reason, although doing global optimization before local optimization is more intuitive, in all the remaining experiments, the proposed optimization framework has been configured in the other way around, running the Petrophysical Optimizer before the Global Optimizer.

**Fig 8:**
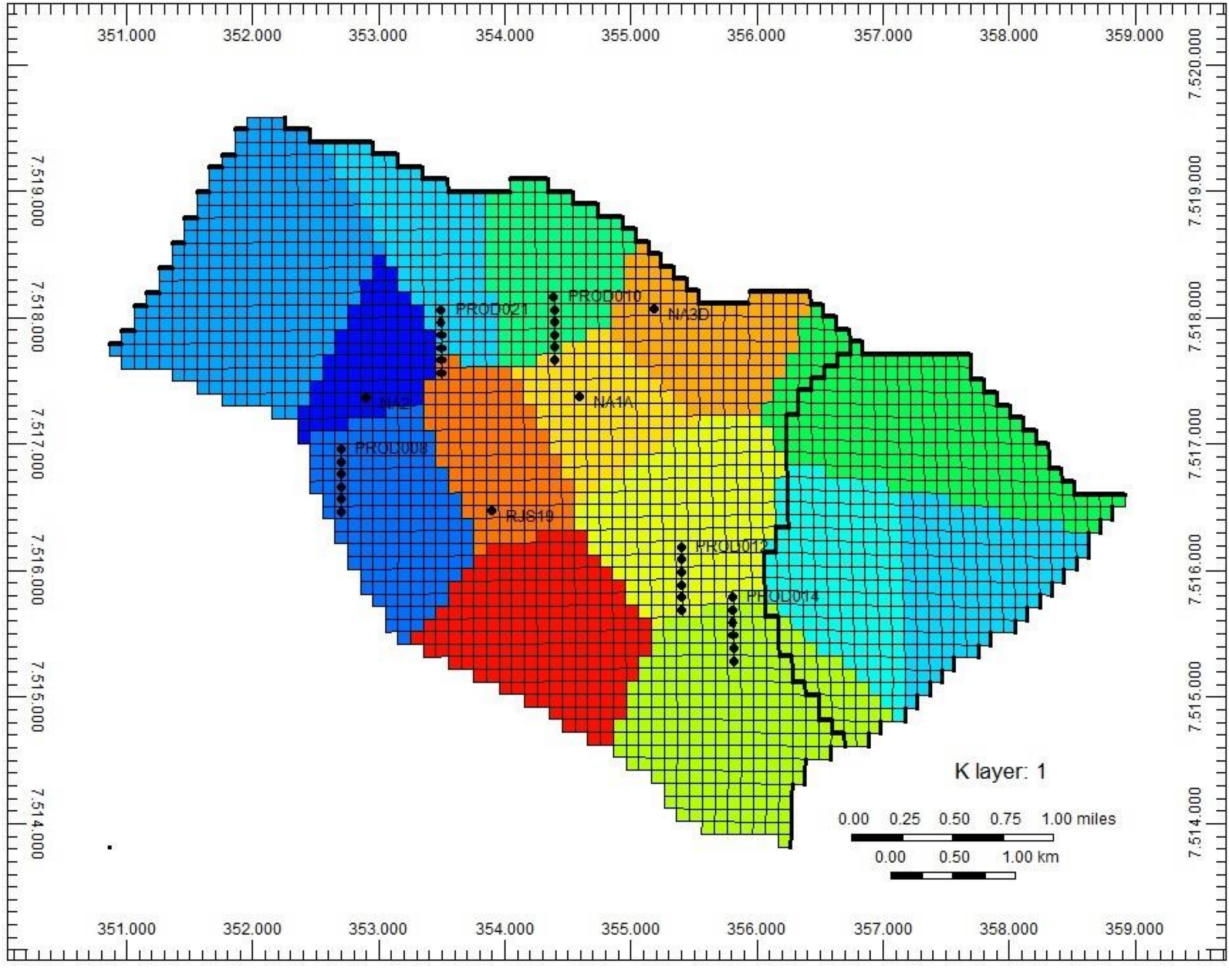
Example of the regions obtained with the Regions Definition algorithm during the execution of the optimization framework with UNISIM-I-H-A S1 scenario.

The second preliminary experiment intended to verify the shape of the reservoir regions identified by the “Regions Definition” strategy (Section 2.4.1). Fig 8 shows an example of the regions obtained during the execution of the optimization framework with the UNISIM-I-H-A S1 scenario. The proposed clustering strategy does not necessarily ensure that blocks in the same spatial pattern of geological structure will be grouped in the same region—what may not even be feasible given the requirement of having one single well per region. However, looking at Fig 8, it can clearly be seen that the resulting regions respect almost perfectly the geological structure of the reservoir sealing fault represented by the black line. This is an indicative that the proposed “Regions Definition” strategy is really an interesting approach to partition the reservoir grid into regions: it allows grouping blocks spatially closed to each other but also with similar values of petrophysical properties, and very likely within the same spatial pattern of geological structure.

**Fig 9:**
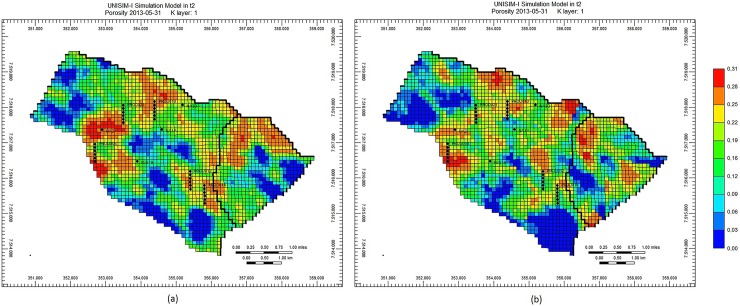
Porosity on initial best solution (a) and on final best solution (b), scenario UNISIM-I-H-A S1.

**Fig 10:**
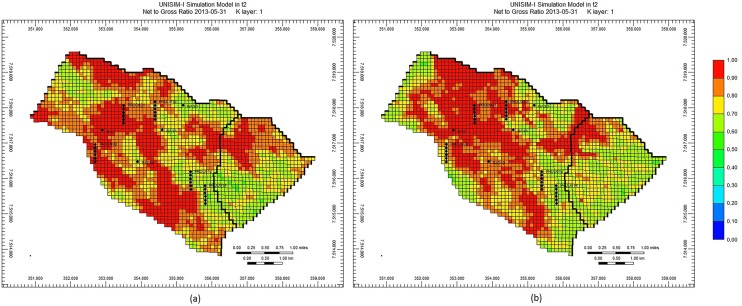
Net to Gross Ratio on initial best solution (a) and on final best solution (b), scenario UNISIM-I-H-A S1.

**Fig 11:**
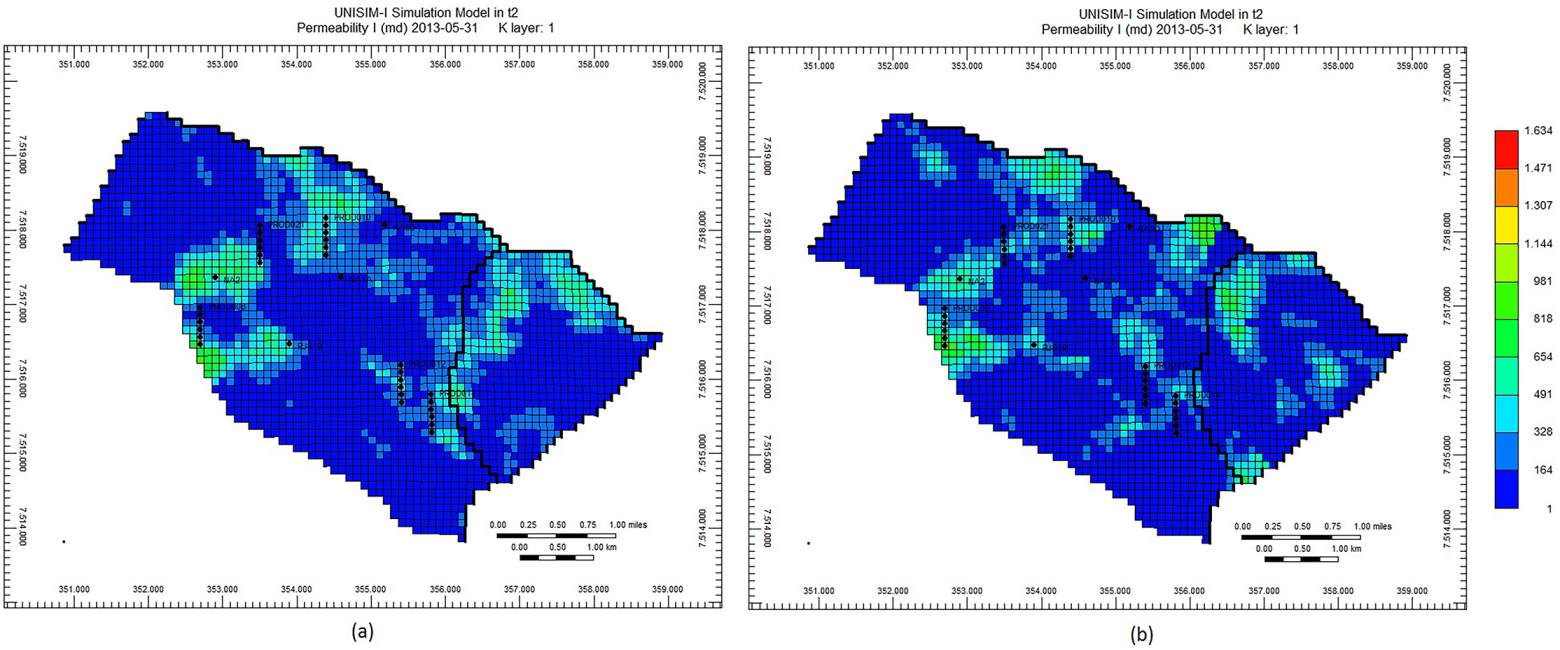
Permeability I on initial best solution (a) and on final best solution (b), scenario UNISIM-I-H-A S1.

The final preliminary experiment was made with the purpose of verifying the petrophysical images generated by the Petrophysical Optimizer (Section 2.4). Maintaining the geological consistency, respecting the spatial correlation of petrophysical properties, is indeed an important aspect to be considered in a history matching strategy. Fig 9, Fig 10 and Fig 11 depict, for the UNISIM-I-H-A S1 scenario, the images of three petrophysical properties (Porosity, Net to Gross Ratio and Permeability I, respectively) in the initial best solution and in the final best solution obtained at the end of the execution of the proposed optimization framework. These images show that, although geological realism has not been a primary concern of the optimization framework, the strategy of the Petrophysical Optimizer generates petrophysical images that are definitely not anomalies from a geological point of view. The initial images are all geological consistent (since they came from the UNISIM-I-H characterization), and, as can be clearly seen in Fig 9, Fig 10 and Fig 11, the images generated by the Petrophysical Optimizer have a very similar geological structure.

### 3.5 Experiment with the complete optimization framework on UNISIM-I-H-A dataset

**Fig 12:**
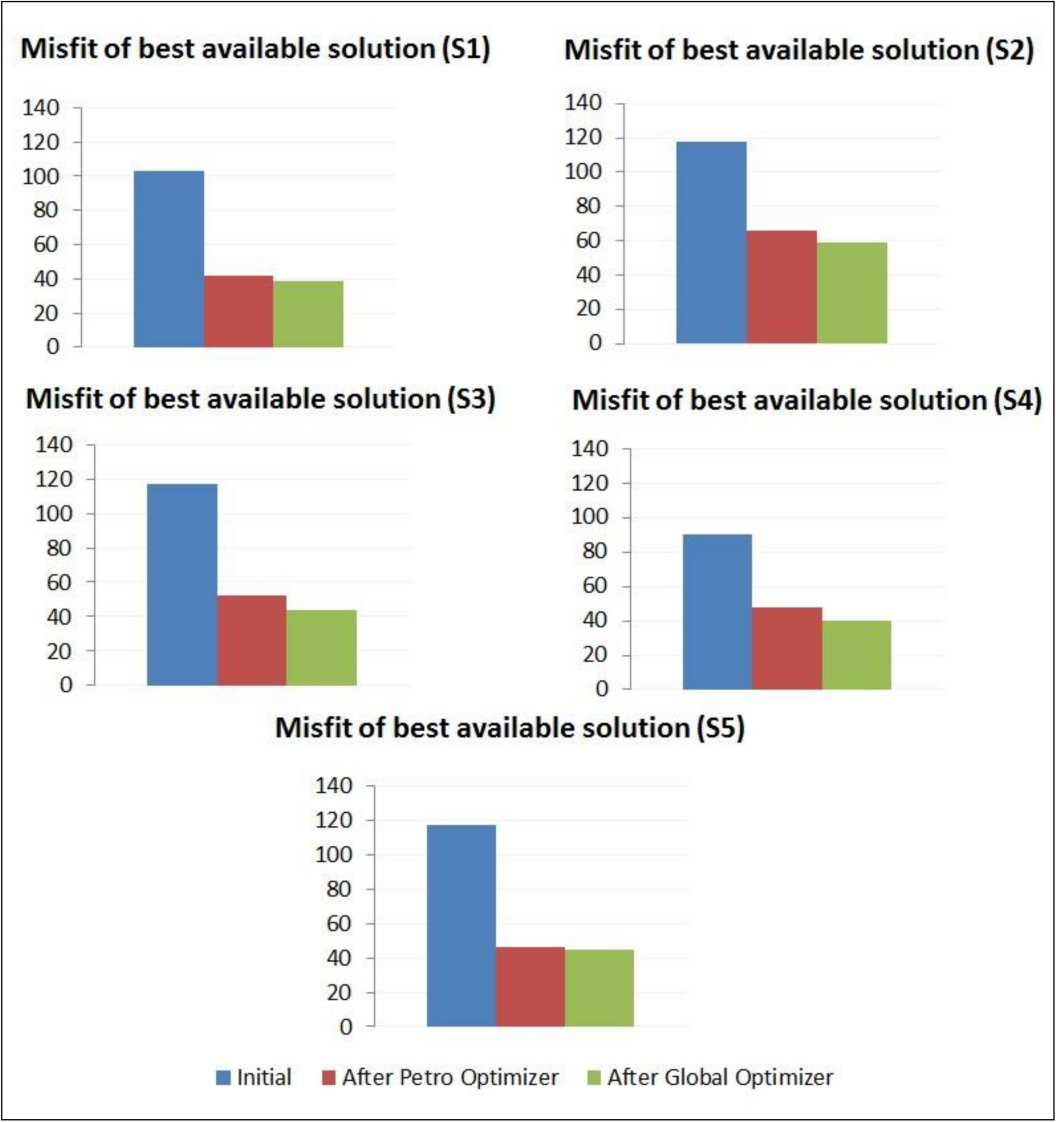
Evolution of the misfit values of best available solution during the optimization framework execution.

In this experiment, the performance of the implemented dynamic decision-making framework was evaluated on dataset UNISIM-I-H-A (Section 3.2.1). Fig 12 shows the evolution of the misfit value of the best available solution for each scenario in the dataset and Table 9 summarizes the corresponding improvements and the total number of simulations (including the 100 of the initial models) executed in each case.

**Table 9:** Improvements on the misfit values of best available solutions and total number of simulations during the optimization framework execution.

Scenario	Improvement (%) after Petrophysical Properties Optimizer	Improvement (%) after Global Properties Optimizer	Total Improvement (%)	Total #of Simulations
S1	59.8	6.6	62.5	190
S2	44.0	10.9	50.1	185
S3	55.2	16.6	62.6	175
S4	47.0	15.9	55.4	185
S5	60.3	4.8	62.2	165

The results show that the Petrophysical Properties Optimizer component was the responsible for the highest portion of the improvements. It can also be noted that, for all the scenarios in UNISIM-I-H-A dataset, the total improvement was higher than 50% and, on average, the misfit of the initial best solution has been improved 58.6% during the optimization framework execution.

**Fig 13:**
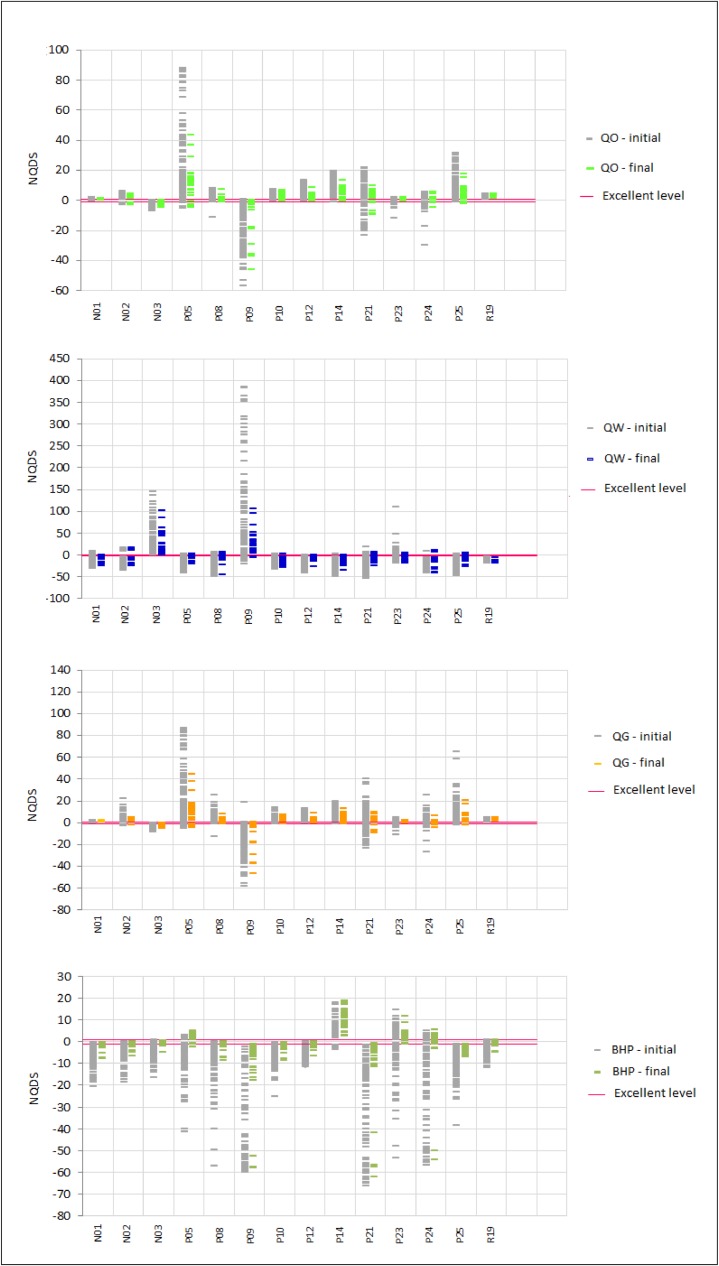
NQDS graphics for producer well series in scenario S1.

**Fig 14:**
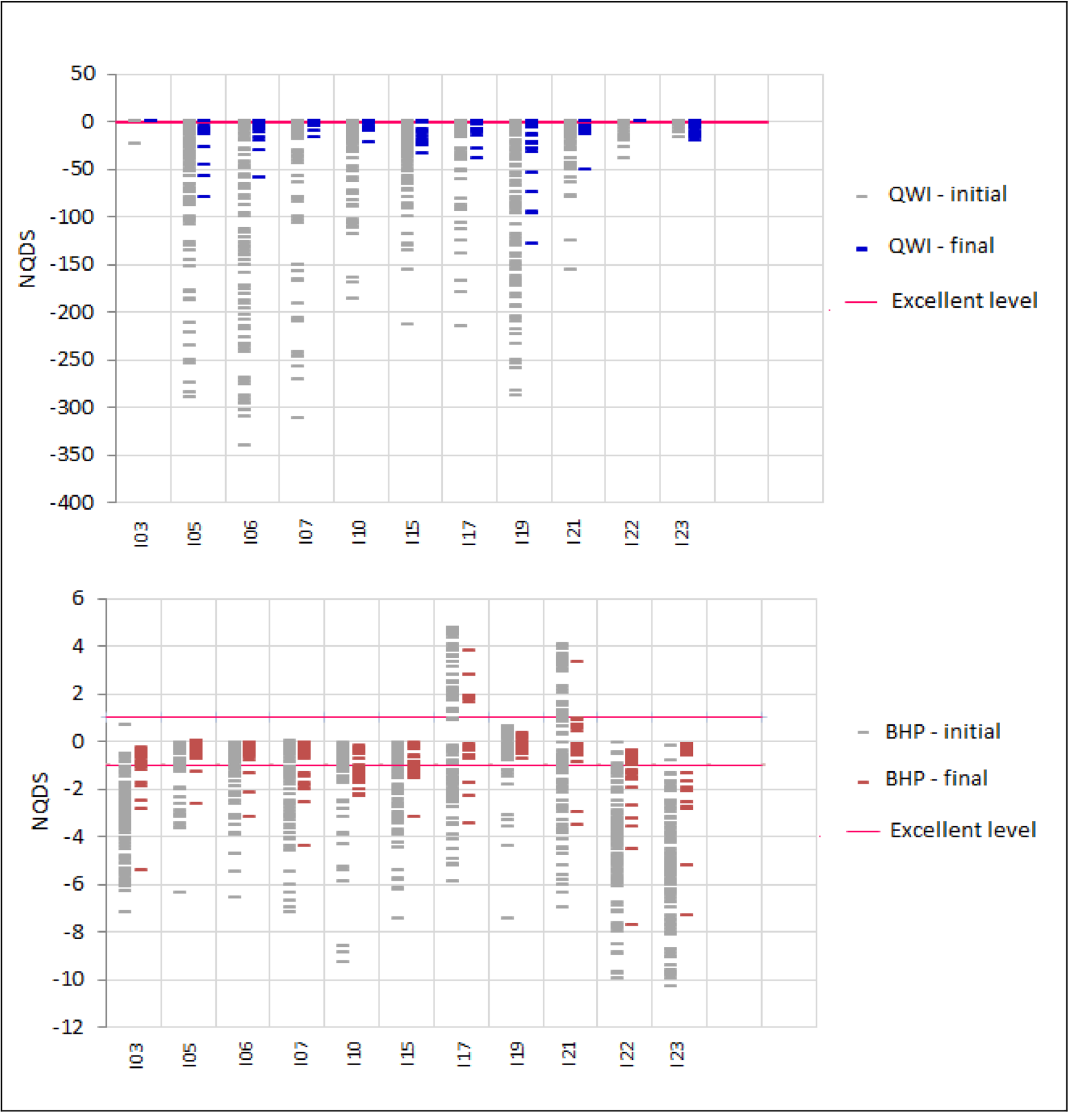
NQDS graphics for injector well series in scenario S1.

Fig 13 and Fig 14 show, respectively, the NQDS graphics for the producer and injector well series in scenario S1. The graphics for the other scenarios from UNISIM-I-H-A dataset were similar to the ones presented in these two figures.

The NQDS graphics are a more compact way of presenting the results of a history matching process. While a production history match plot may be necessary for specific analyses, a single NQDS plot can be used to visualize simultaneously the data of several variables. In the case of UNISIM-I-H benchmark, which involves the matching of 78 variables, without the NQDS graphics of Fig 13 and Fig 14, it would have been necessary to generate 78 production plots to evaluate and present the same results.

Each column in the NQDS plots of Fig 13 and Fig 14 represents the history matching quality of a particular variable, for a set of models, and may be interpreted in the following way: the more the column fits inside the region delimited by the red horizontal lines, which represent the “Excellent” quality range, the better is the history matching quality of the particular variable in the considered models.

In Fig 13 and Fig 14, the columns labeled with ‘-initial’ show the NQDS values for the 100 initial models in S1 scenario. Similarly, the columns labeled with ‘-final’ show the NQDS values for the 100 best models after the optimization framework execution. It can be clearly seen from the NQDS plots of these two figures that the ‘-final’ columns are closer to the excellent range (red horizontal lines) for all series considered in the history matching process. This result shows how the proposed optimization framework can indeed improve the NQDS values towards the excellent quality level, and hence find models with better history matching quality.

**Fig 15:**
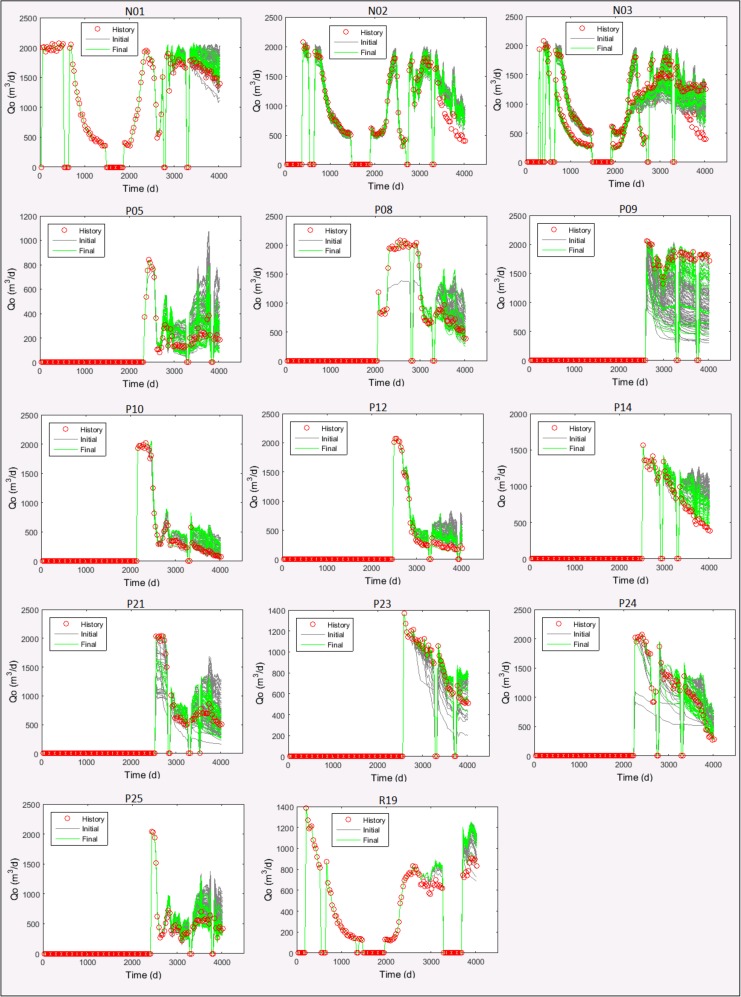
Oil rate curves for the producers.

In order to illustrate the correspondence between a column in an NQDS plot and a set of curves in a production history matching plot, consider the NQDS plot for the oil rate (QO) of all producers in Fig 13. In this plot, the grey columns (QO-initial) show the NQDS values for QO, in the 100 initial models. The green columns (QO-final) show the NQDS values for QO, in the 100 final best models obtained with the execution of the proposed framework. Now, consider the oil production rate curves, in Fig 15. These curves were generated with the same models used in Fig 13. The grey curves are the ones corresponding to the 100 initial models in scenario S1, and the green ones are the ones which correspond to the 100 final best models obtained with the framework execution. Note, for example, that the green columns for N01, N02, and N03 wells in the QO NQDS plot in Fig 13 are just slightly better than the corresponding grey columns. The same behavior is verified for the oil rate curves of these wells in Fig 15: the green curves are just slightly closer to the history curve. On the other hand, the green columns of wells P5, P12, P14, P21, and P25 in the QO NQDS plot of Fig 13 clearly outperform the corresponding initial grey columns. The oil rate curves of these wells, in Fig 15, confirm this fact: the green curves are much closer to the history curve.

**Fig 16:**
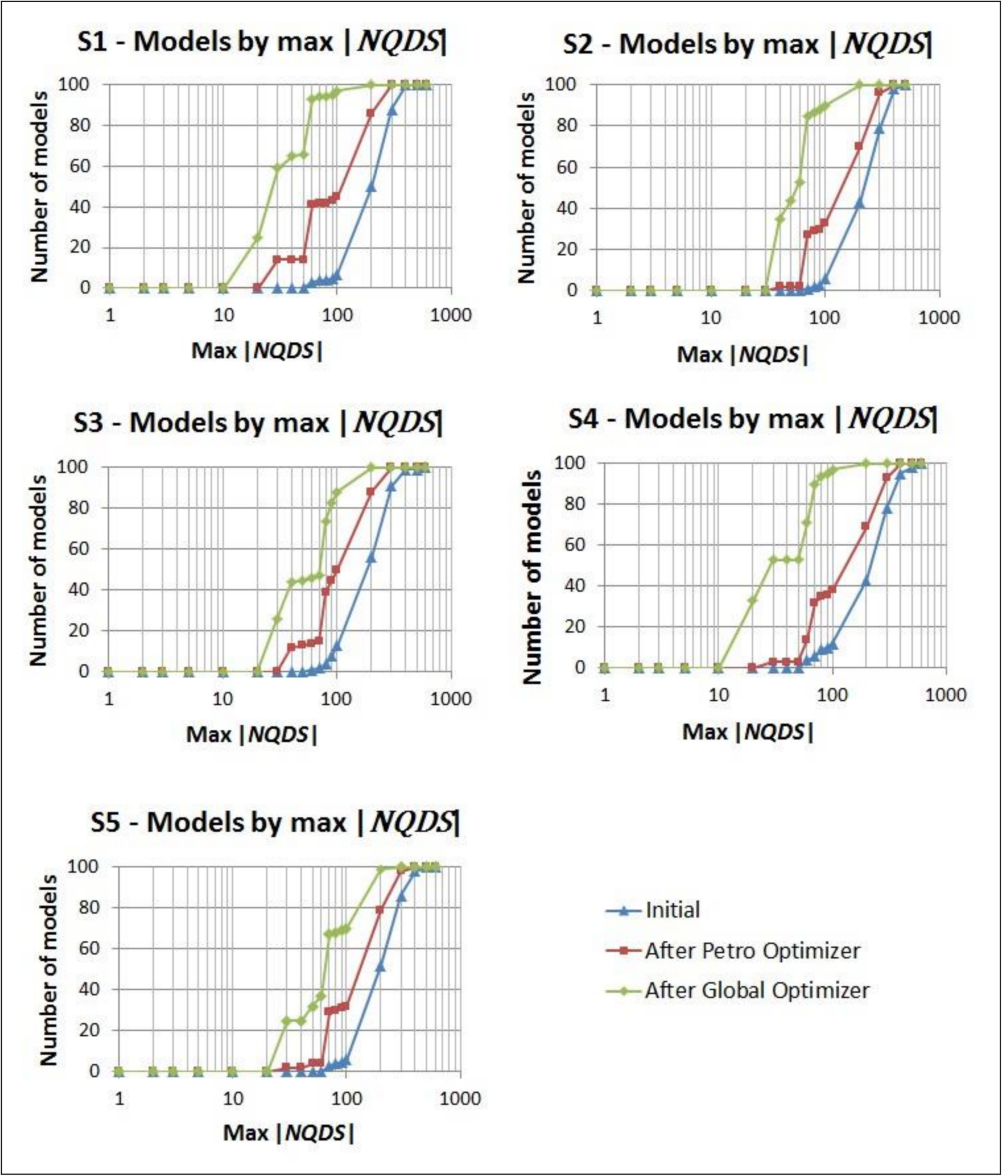
Distribution of models by maximum |*NQDS*_*x*_ |, for the 100 best models, during the optimization framework execution using UNISIM-I-H-A dataset.

Fig 16 shows the evolution on the number of models by maximum |*NQDS*_*x*_|, for the 100 best models, during the optimization framework execution. The ideal output of a history matching process, considering the quality levels defined in Table 1., would be having the final curves (the green ones in the graphics of Fig 16) starting as much vertical, and close to 1, as possible. Although this ideal has not been reached here, the output of the optimization framework in all the scenarios in UNISIM-I-H-A dataset clearly shows that the number of models with lower values of maximum | *NQDS*_*x*_ | has increased during the framework execution.

**Fig 17:**
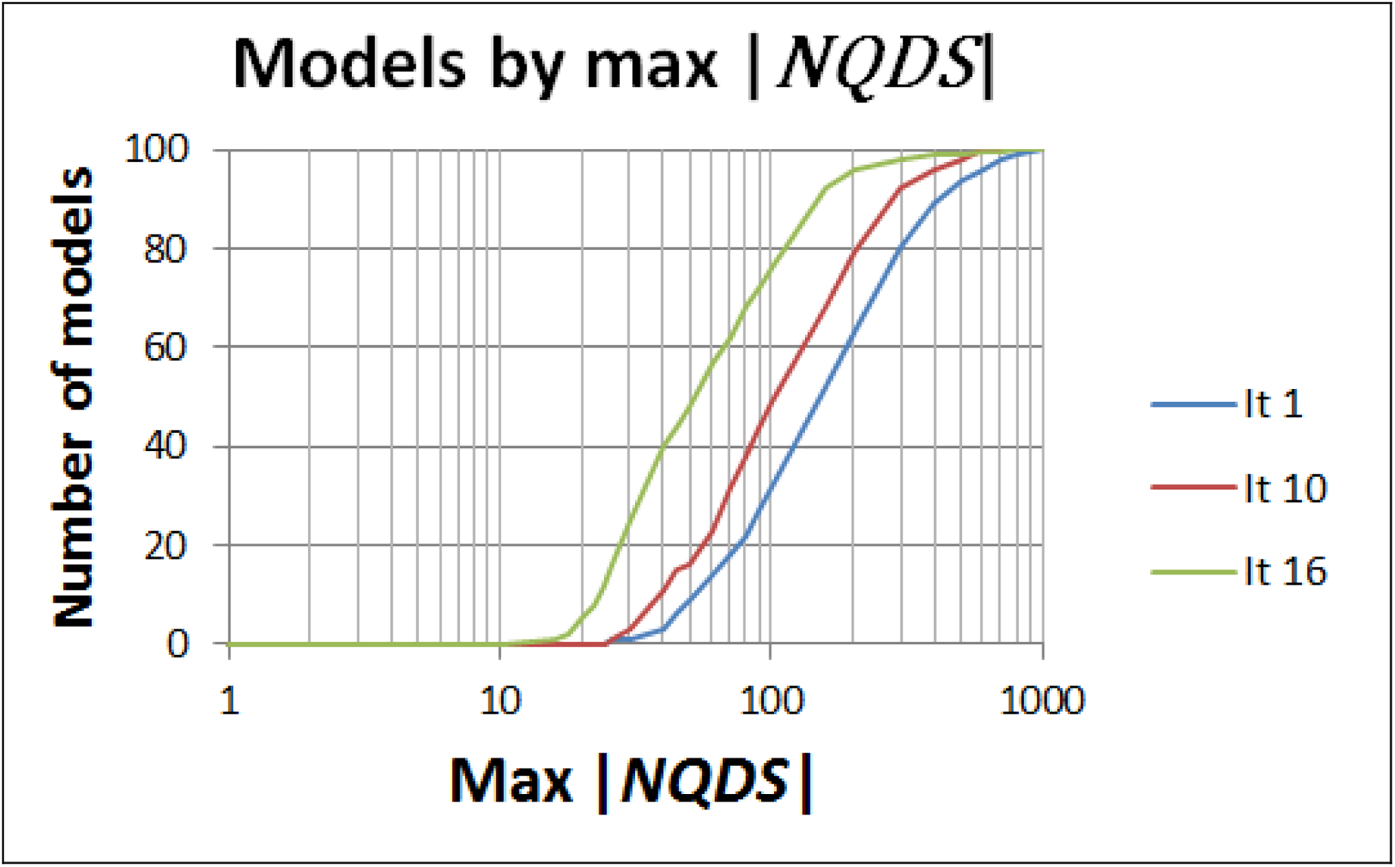
Distribution of models by maximum |*NQDS*_*x*_ |, based on the results reported by Mesquita et al. [8].

Fig 17 shows the results reported by Mesquita et al. [8] for a scenario based on the same UNISIM-I-H benchmark. Comparing the final 100 best models obtained with the proposed optimization framework (Fig 16) with the ones reported by Mesquita et al. [8] (Fig 17), the following points are worth mentioning:

1. The decrease on the maximum |*NQDS*_*x*_ | is larger with the proposed optimization framework. In all the scenarios from UNISIM-I-H-A dataset, the final green curves in Fig 16 are more vertical than the final one (Iteration 16) reported by Mesquita et al. [8] (Fig 17). Also, in four, out of five, scenarios of UNISIM-I-H-A dataset, at least 88% of the 100 final best models generated with the proposed optimization framework have |*NQDS*_*x*_ | ≤ 100. This number contrasts with the corresponding 75–78% reported by Mesquita et al. [8].
2. Neither the proposed optimization framework nor the strategy presented by Mesquita et al. [8] was able to generate models with maximum | *NQDS*_*x*_ | ≤ 10. This is a sign of how difficult is the history matching problem of the UNISIM-I-H benchmark and reinforces that efforts to improve the current solutions are desirable and important.
3. The total number of simulations used by the proposed optimization framework was much smaller: none of the scenarios from UNISIM-I-H-A dataset used more than 190 simulations (Table 9), which is about 9% of the 2,100 simulations used by Mesquita et al. [8].

### 3.6 Experiment with the petrophysical optimizer on UNISIM-I-H-A and UNISIM-I-H-B datasets

**Fig 18:**
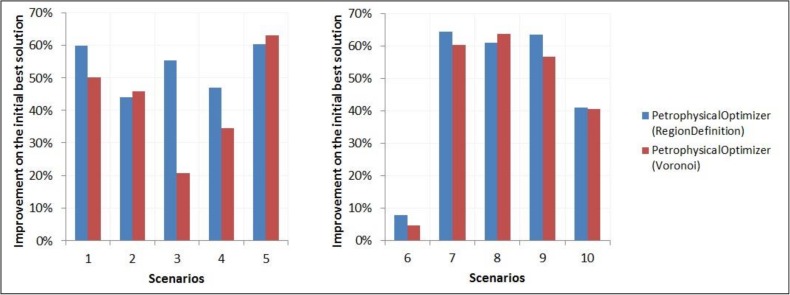
Improvements on the misfit value of best available solution obtained with the two versions of the petrophysical properties optimizer, on the scenarios of UNISIM-I-H-A and UNISIM-I-H-B datasets.

This experiment aimed at evaluating the improvements on history matching solutions obtained with the petrophysical properties optimizer component (Section 2.4). The proposed component, using the “Regions Definition” strategy (Section 2.4.1) for partitioning the reservoir into regions, was compared against a modified version in which the “Regions Definition” step was replaced by a partitioning algorithm using the traditional Voronoi approach to delimit the regions around the reservoir wells. Fig 18 compares the improvements on the initial best solution obtained with the original optimizer with the corresponding ones obtained with the modified optimizer, for the scenarios in UNISIM-I-H-A and UNISIM-I-H-B datasets.

From the results in Fig 18, one can see that, for UNISIM-I-H-A dataset, in three, out of five, scenarios (S1, S3 and S4), the improvements obtained with the version of the petrophysical properties optimizer using the Region Definition step was largely superior when compared with the corresponding improvements obtained with the Voronoi partitioning algorithm. For the remaining two scenarios, S2 and S5, where the use of the Voronoi partitioning strategy led to better results, the difference was below 5%. For the UNISIM-I-H-B dataset, the improvements obtained with the version of the petrophysical properties optimizer using the “Regions Definition” step were superior in four, out of the five, scenarios.

**Fig 19:**
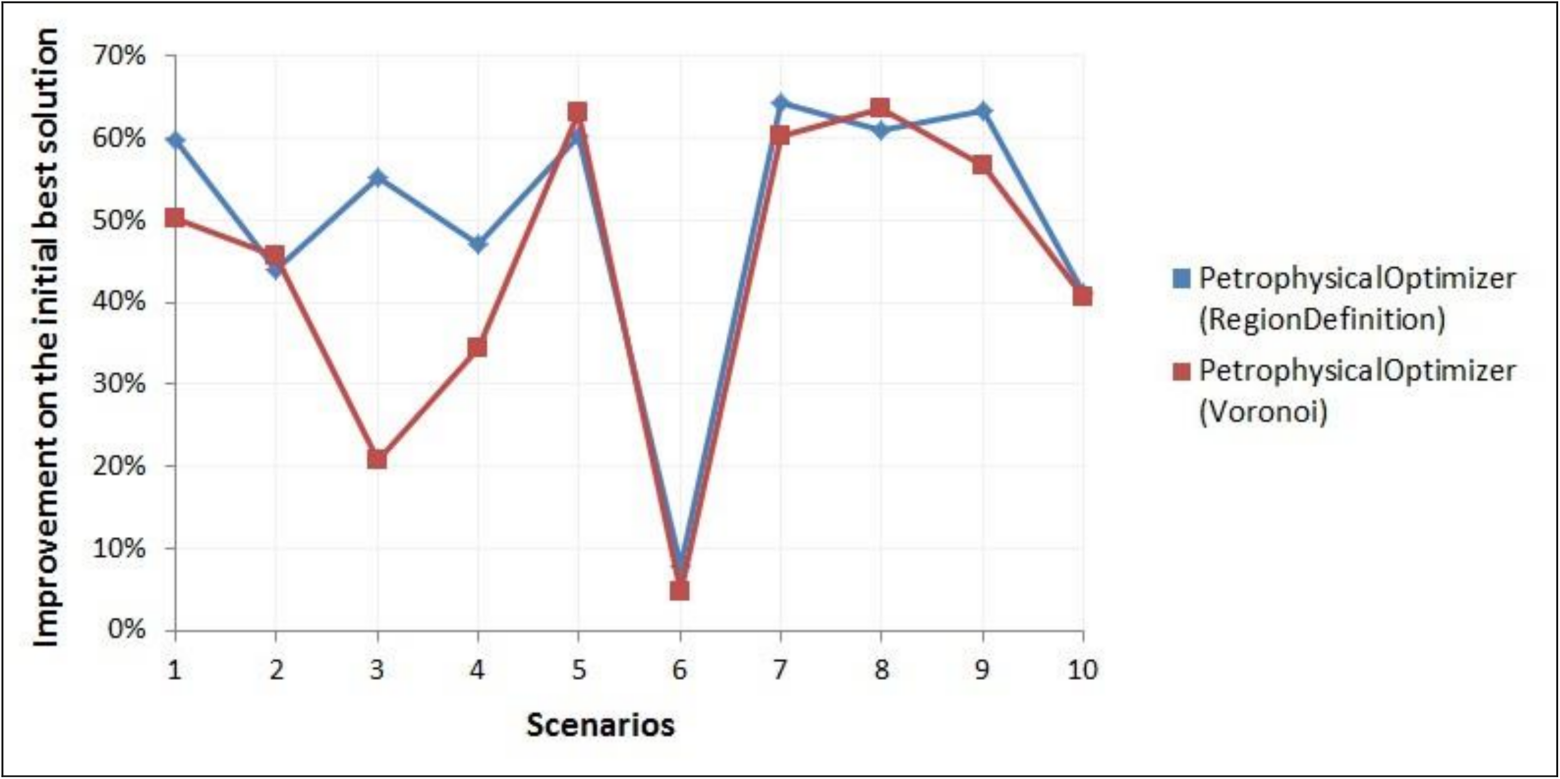
Improvements on the misfit value of best available solution obtained with the two versions of the petrophysical properties optimizer, on scenarios from UNISIM-I-H-A and UNISIM-I-H-B datasets.

Fig 19 depicts, for the 10 scenarios from UNSIM-I-H-A and UNISIM-I-H-B datasets, the comparison of the improvements on the misfit value of the best available solution obtained with the two versions of the Petrophysical Properties Optimizer. A two-sample paired Wilcoxon signed rank test [15] conducted on these data led to a p-value of 0.06. With this result, we can conclude that the two versions of the Petrophysical Optimizer are, with 90% confidence, statistically different.

## 4 Conclusions

This paper presented a dynamic decision-making optimization framework for history matching problems. The proposed framework comprises two optimization components: the first one focused on changes of petrophysical properties and the second one with the purpose of improving the history matching solutions through the change of global reservoir properties.

The proposed framework has been applied to the UNISIM-I-H benchmark and the results show the potential of the framework in improving the quality of history matching solutions. Indeed, in terms of the final 100 best models found, the improvements obtained with the framework were larger than previous improvements reported by Mesquita et al. [8] for the same benchmark and were achieved using only 9% of the number of simulations.

The proposed Petrophysical Properties Optimizer component introduced a new strategy to partitioning the reservoir into distinct regions. The results obtained with the new strategy were superior to the ones obtained with the traditional Voronoi approach in 60% of the dataset scenarios used in the experiments.

The combination of the proposed Petrophysical Properties Optimizer with the Global Properties Optimizer, both using information learned from the dynamic evaluation of the available data, could clearly improve the initial quality of history matching solutions. This shows how the use of machine learning techniques, as the ones adopted in this work, can be promising in the context of a history matching process.

Results obtained with the dynamic decision-making approach presented in this paper indicate it may indeed be an option to improve the quality of history matching solutions with a small number of simulations. Also, the proposed strategy to partitioning the reservoir into distinct regions, based on the k-means clustering algorithm, is an alternative for the traditional Voronoi partitioning as it may lead to better results.

Future work includes extending the dynamic decision-making optimization framework with other components so that better history matching solutions can be achieved. Optimizer components that use soft clustering algorithms to partitioning the reservoir regions or that dynamically focus on the matching of well variables having high misfit are some ideas that can be explored and may bring further improvements in the search of history matching solutions with high quality.

## Supporting information

S1 Appendix Walk through the basic concepts of a history matching problem. (PDF)

S1 File Mesquita et al 2015. A Systematic Approach to Uncertainty Reduction with a Probabilistic and Multi-Objective History Matching.
(PDF)
