# Supplementary material for: History matching through dynamic decision-making
Source: PLoS One. 2017 Jun 5;12(6):e0178507. doi: 10.1371/journal.pone.0178507 (PMC5459344; doi:10.1371/journal.pone.0178507)
Supplement: S1 Appendix — (PDF) [file pone.0178507.s001.pdf]

Walk through the Basic Concepts of a History Matching Problem

This Appendix illustrates the main concepts involved in the history matching process using the scenario shown in Fig A. 1.

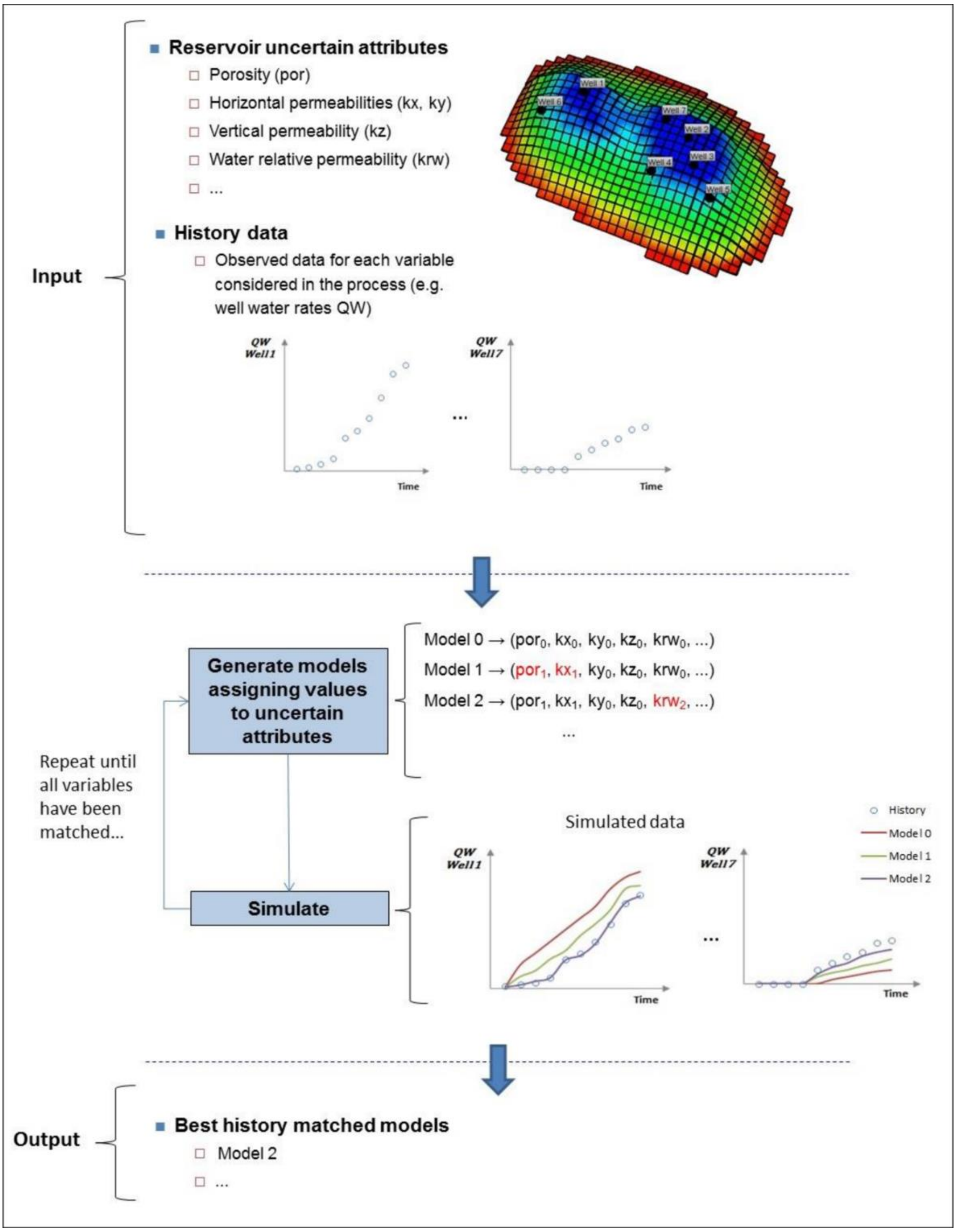

Fig A. 1 - Illustration of a typical history matching process.

The process input is the reservoir uncertain attributes and the history data that need to be matched. The uncertain attributes vary with the reservoir model being adjusted and, in general, can be classified into two categories: global attributes, which values impact the reservoir performance as a whole; and local attributes which values are associated with a particular reservoir area, as for example, a block of the reservoir model grid or a specific region defined around one of the reservoir wells.

The history data corresponds to the real reservoir performance information and comprises curves, overtime, for each variable that needs to be matched. Common variables considered in a history matching process are the pressure and dynamic production data (oil, water and gas rates) of the reservoir wells.

The core of the history matching process is the generation of new models using different values of the uncertain attributes. What uncertain attribute needs to be changed and to which value it needs to be set are some of the questions that arise during the process. As exemplified in in Fig A. 1, new models can be generated changing just one attribute at a time, or changing many attributes at once. Moreover, the uncertain attributes are very often correlated and even changes in local attributes may impact neighbor areas, due to the intrinsic communication between the different reservoir regions.

During the history matching process, each new model generated needs to be simulated so that the model performance, for each variable, can be compared with the history data.

In the example of Fig A. 1, the history matching process involves only seven variables (the water rates of each well) and it would finish returning models such as “Model 2” which has, for the variables considered in the process, a performance very similar to the history reservoir data.
