# Supplementary material for: History matching through dynamic decision-making
Source: PLoS One. 2017 Jun 5;12(6):e0178507. doi: 10.1371/journal.pone.0178507 (PMC5459344; doi:10.1371/journal.pone.0178507)
Supplement: S1 File — A Systematic Approach to Uncertainty Reduction with a Probabilistic and Multi-Objective History Matching. (PDF) [file pone.0178507.s002.pdf]

Th N117 06

A Systematic Approach to Uncertainty Reduction with a Probabilistic and Multi-Objective History Matching

F.B. Mesquita* (Universidade Estadual De Campinas), A. Davolio (Universidade Estadual De Campinas) & D.J. Schiozer (Universidade Estadual De Campinas)

SUMMARY

The petroleum industry has a high demand for production forecasting under uncertainties, which is performed through probabilistic approaches. Although these approaches have been used very often in recent years, one of the challenges to their application is the development of a methodology with multiple scenarios that honor the dynamic data available (history matching procedures). Many works, which propose different probabilistic history matching approaches have been published in the literature but are based on methods that demand a very high number of reservoir simulation runs. Thus, to make this type of application feasible, these methods rely on the application of proxy models, which, however, are not able to capture the full physical behavior of the model. Different from those methods, this work proposes a methodology for uncertainties reduction of reservoir properties integrated with an assisted history matching procedure, in a multi-objective approach that does not employ proxies.

SPE-174359-MS

A Systematic Approach to Uncertainty Reduction with a Probabilistic and Multi-Objective History Matching

F.B. Mesquita, A. Davolio, and D.J. Schiozer, Universidade Estadual de Campinas

Copyright 2015, Society of Petroleum Engineers

This paper was prepared for presentation at the EUROPEC 2015 held in Madrid, Spain, 1–4 June 2015.

This paper was selected for presentation by an SPE program committee following review of information contained in an abstract submitted by the author(s). Contents of the paper have not been reviewed by the Society of Petroleum Engineers and are subject to correction by the author(s). The material does not necessarily reflect any position of the Society of Petroleum Engineers, its officers, or members. Electronic reproduction, distribution, or storage of any part of this paper without the written consent of the Society of Petroleum Engineers is prohibited. Permission to reproduce in print is restricted to an abstract of not more than 300 words; illustrations may not be copied. The abstract must contain conspicuous acknowledgment of SPE copyright.

Abstract

The petroleum industry has a high demand for production forecasting under uncertainties, which is performed through probabilistic approaches. Although these approaches have been used very often in recent years, one of the challenges to their application is the development of a methodology with multiple scenarios that honor the dynamic data available (history matching procedures). Many works, which propose different probabilistic history matching approaches have been published in the literature but are based on methods that demand a very high number of reservoir simulation runs. Thus, to make this type of application feasible, these methods rely on the application of proxy models, which, however, are not able to capture the full physical behavior of the model. Different from those methods, this work proposes a methodology for uncertainties reduction of reservoir properties integrated with an assisted history matching procedure, in a multi-objective approach that does not employ proxies.

The methodology proposed is an extension of a procedure previously published by our group, which has shown to be an efficient way to perform probabilistic history matching. At each iteration of the process, the model uncertainties are combined through a robust sampling technique, which originates a set of model realizations that are then simulated. The results are evaluated for all Objective Functions (OF), namely, the quadratic deviations of all well rates and pressures. This work proposes the use of quantitative tools to perform the matching: (1) indexes that quantify the matching quality for each OF and allow prioritizing the worst OF to be matched and (2) a bi-dimensional matrix that supports the identification of sources to the highest deviations and gradual model properties updating. At the end of the procedure, good models (which must present satisfactory matching for all OF) are filtered and further employed in production forecasting under uncertainties.

The methodology is applied to a synthetic model (UNISIM-I-H) based on the Namorado field in Brazil. The results show not only the gradual improvement of all OF during the matching procedure but also and, more importantly, how the reservoir uncertainties are updated based on the tools proposed here.

The proposed indicators make the application systematic and can be used for automatizing the process. The methodology is able to deal with complex cases, especially those involving several wells and uncertainties with non-linear interaction among them. The developments provide robustness to the application, resulting in a more reliable production forecast and management of the field.

Introduction

Understanding the reservoir behavior has always been of fundamental importance for the petroleum industry. It involves the reservoir characterization, which is a multi-disciplinary topic, and is responsible for the serious challenge of populating the reservoir model with sparse data. All decisions in the life cycle of a petroleum field involve high investments or high incomes, so robust reservoir models are required to adequately represent the fluid flow. In order to achieve robust models, dynamic data, measured at the wells during some period of reservoir production, are used to calibrate the model in a procedure called history matching.

History matching is an inverse process in which the reservoir properties are modified in an attempt to honor all the available dynamic data, namely, well production (or injection) rates, well pressures and 4D seismic data. Although employed for several years, its application still faces important problems. One of them is the non-unique solution of the inverse problem, in which the output data (reservoir response) is known and the input (reservoir properties) is not. One way to mitigate this issue is through the use of probabilistic methods: the model properties to be adjusted are treated as uncertainties and, at the end of the process, the production forecasting is carried out for reservoir scenarios under uncertainties.

Recently, several stochastic optimization methods to quantify uncertainty with the incorporation of production data have been proposed. Different approaches can be mentioned: the Stochastic Gaussian Search Direction (SGSD) algorithm (Li and Reynolds 2011), the evolutionary algorithm (Al-Shamma and Teigland 2006), the Bayesian optimization algorithm (Abdollahzadeh et al. 2011), the ant colony optimization (Hajizadeh et al. 2011), and the particle swarm optimization method (Martínez et al. 2012). Most of these methods require a high number of numerical simulations, so, in order to make their application computationally feasible, they commonly simplify the physics of the reservoir model by representing them with proxy models.

Maschio et al. (2005) published one of the first applications of history matching integrated with uncertainty reduction by employing numerical simulation, without considering proxies. They proposed an assisted approach, allowing an iterative process in which the models were generated by combining the uncertain properties through the derivative tree technique. The methods to change the discretized uncertainties were probability redistribution, level elimination, and redefinition of attribute values. Becerra et al. (2008), Maschio et al. (2009) and Becerra et al. (2011) presented some advances of this methodology by applying it to more complex reservoir models, and showed the benefits of considering uncertainties in the production forecasting.

Risso et al. (2011) compared the precision of simulation results for risk analysis applications by using different techniques for dealing with reservoir uncertainties and generating model combinations. They showed that the Latin Hypercube (LH) was the best option when considering precision and number of simulations. Maschio et al. (2010) proposed a sampling algorithm for probability redistribution combined with the use of the LH in order to deal with complex cases with a large number of attributes.

Almeida et al. (2014) introduced a new manner of diagnosing the models' deviations by considering all history data from the wells in a multi-objective approach. The deviations from history were evaluated through a normalized quadratic function for each well data series and analyzed in a concise plot, aiming to identify global and local features to support changes to model properties in an iterative process. The models were generated by combining the uncertainties through Discretized Latin Hypercube with Geostatistical realizations (DLHG) (Schiozer et al. 2015), which attempts to combine the efficiency of the LH sampling technique with the geological consistency of geostatistical methods.

Avansi and Schiozer (2015) demonstrated the importance of integrating reservoir characterization with reservoir simulation studies. They created a synthetic reservoir model with real characteristics (UNISIM-I model) and tested different methodologies for probabilistic history matching by employing a similar approach to the one used in Almeida et al. (2014). In their workflow, all types of modifications to the

model uncertainties were considered: (1) change to the simulation model if the numerical model was considered inadequate to represent the flow phenomena in the reservoir, (2) reparameterization in which the original range limits of uncertainties were modified or a new uncertainty was included or, depending on the case, new geostatistical images were generated or updated and (3) change to probability of uncertain attributes, including the levels elimination.

Objective

This work is an extension of the previous works developed by Avansi and Schiozer (2015) and Almeida et al. (2014). The same probabilistic and multi-objective history matching procedure previously established is implemented but in a systematic approach now. The systematization is incorporated into the process through the creation of indicators, with the purpose of supporting and prioritizing modifications to the model uncertainties.

Methodology

Fig. 1 shows the workflow of the methodology, adapted from Avansi and Schiozer (2015). The dashed red box highlights the main contribution of this work: the steps in which the indicators here proposed are applied.

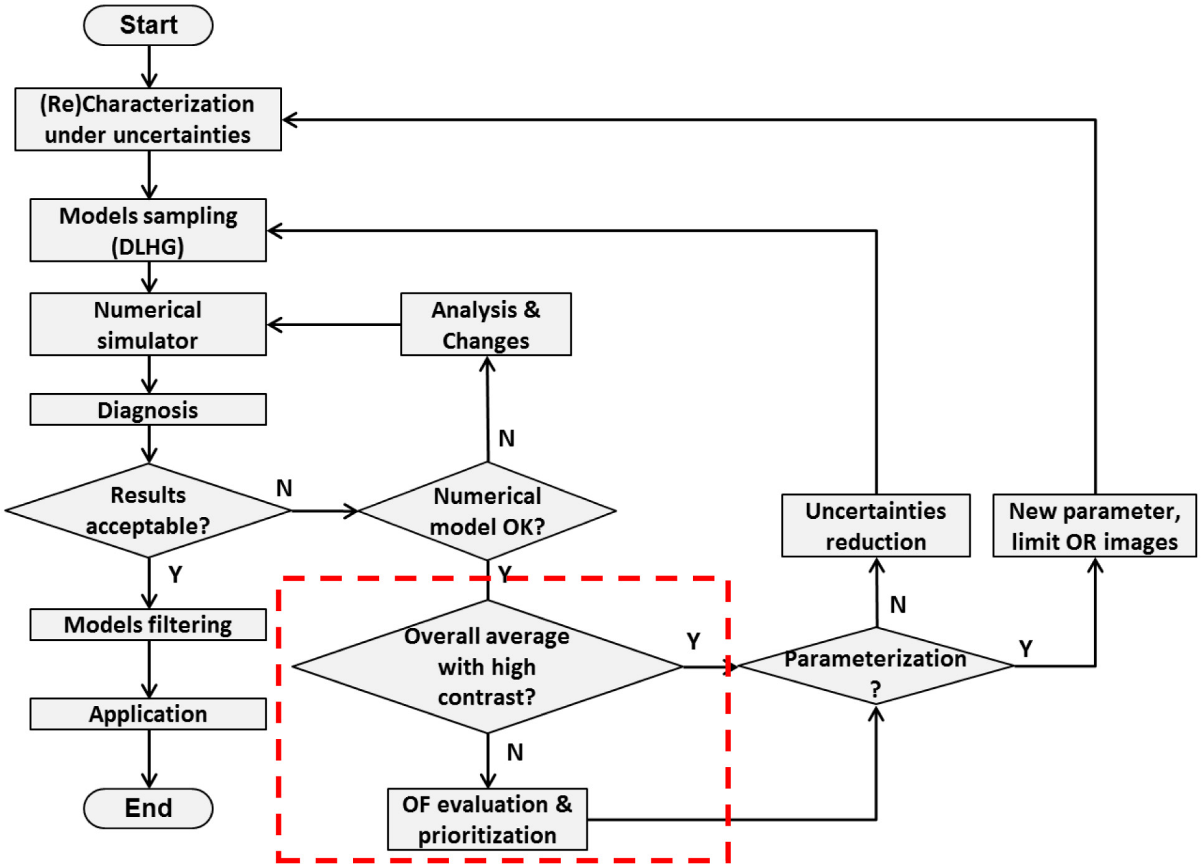

Figure 1—workflow of methodology (adapted from Avansi and Schiozer 2015). Change from the original workflow is highlighted.

The procedure starts by generating several simulation models through a combination of the main reservoir uncertainties using the Discretized Latin Hypercube with Geostatistics (DLHG) method

(Schiozer et al. 2015). The models' deviations from history (data misfits) for all Objective Functions (OF), namely, fluid rates and pressure for all wells, are calculated in the diagnosis phase. If deviations are not acceptable (according to a user defined tolerance), the uncertainty that may be causing the highest deviations is identified, through the Overall Average (OA) detailed below and then updated. When no uncertainty can be identified as the cause of high deviations for several OF, the misfits for an individual OF are evaluated by prioritizing the OF with the worst matching quality. The analysis and modifications of the uncertainties are implemented in a gradual manner. The main steps of the work are detailed as follows.

Models generation and diagnosis

Similar to Almeida et al. (2014), the uncertain attributes, which were previously defined in the model characterization under uncertainty phase, are combined through the DLHG method and the generated model realizations are simulated (first steps of the workflow in Fig. 1).

In the diagnosis step, the history matching quality is evaluated for each well variable (well rates and pressures). The indicator used to measure the deviations between simulated model results and history data is the Normalized Quadratic Deviation with Sign (NQDS), obtained as follows.

$$LD = \sum_{i=1}^{n}(Sim_i - Hist_i), \tag{1}$$

$$QDS = \frac{LD}{|LD|} \times \sum_{i=1}^{n}(Sim_i - Hist_i)^2, \tag{2}$$

$$AQD = \sum_{i=1}^{n}(Tol \times Hist_i + C)^2, \tag{3}$$

$$NQDS = \frac{QDS}{AQD}, \tag{4}$$

where:

LD = linear deviation of the simulated data series from the history data series;
n = number of points for the respective data series;
Sim_i = simulated value at time point "i" for the data series;
$Hist_i$ = history data value at time point "i";
QDS = quadratic deviation with sign of the simulated data series from the history data series;
AQD = acceptable quadratic deviation employed to normalize the NQDS;
C = constant employed to avoid division by zero in Eq. 4 when AQD is zero;
Tol = acceptable tolerance for an OF to be considered good.

The acceptable tolerance (Tol) for an OF to be considered good should be chosen according to the measurement quality of the observed data. For example, if Tol=10% for the Bottom-Hole Pressure (BHP), then NQDS=−1 for a model realization means that the absolute value of QDS is 10% below the history data for this model and, therefore, this model is at the limit of acceptance criteria (Fig. 2).

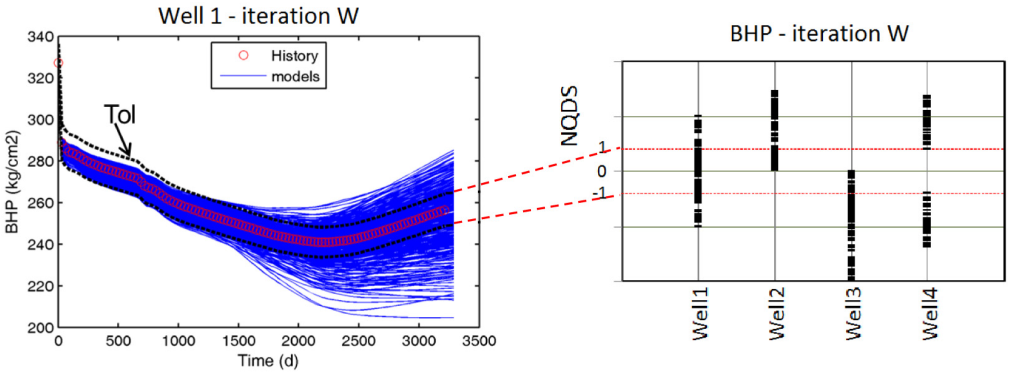

(a) (b)

Figure 2—illustration of how the NQDS plot is built (adapted from Almeida 2014).

After all OF are computed for the simulation results, they are analyzed in order to identify possible model uncertainties causing high models' deviations from history data. In Almeida et al. (2014), this analysis was made based on a plot in which the NQDS for all model realizations was plotted by each well variable (Fig. 2b). This work proposes the use of the Influence Matrix (IM) to analyze the influence from uncertainties on the various OF.

Analysis of data misfit: Influence Matrix and Overall Average

The influence matrix is a bi-dimensional matrix, which permits a full analysis of deviations for each uncertain attribute and for each OF (NQDS of each well variable), simultaneously. As illustrated at the top left in Fig. 3, the NQDS of each well variable are separated by an attribute level (Fig. 3, step1) and the average is computed (step2) for each level. These averages are tabulated (step3) and then inserted in the matrix, each row representing one OF and each column an attribute level. The matrix cells are colored according to their values. In the example presented in Fig. 3, the values inside the acceptance criteria range ($-1 \leq NQDS \leq 1$) are filled with white, the positive values larger than 1, with red and the negative values smaller than -1, blue. The use of colors makes the visualization of deviations for the whole set of models possible, simultaneously for each OF and for each uncertain attribute. This is especially useful for cases that involve several uncertain attributes and a high number of OF.

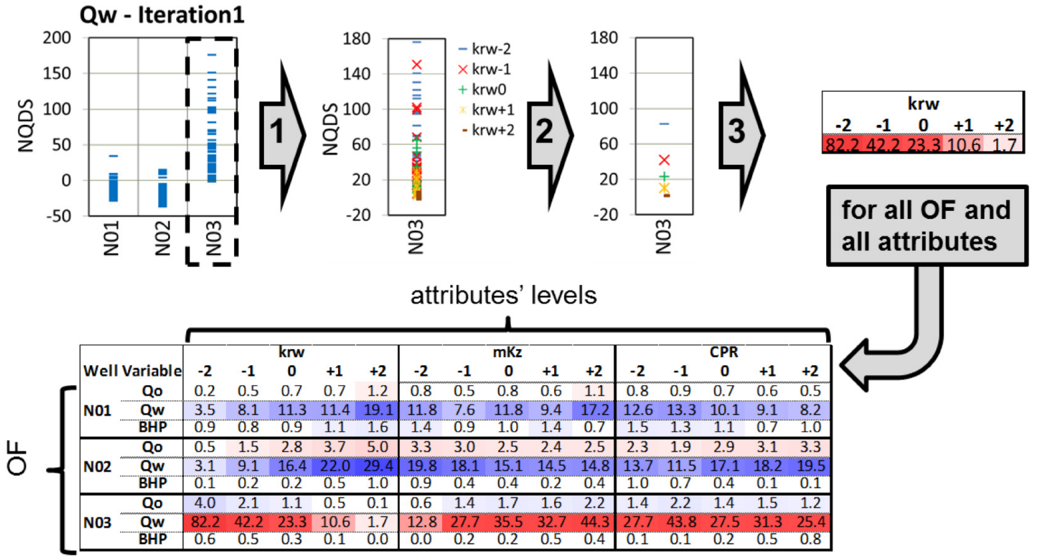

Figure 3—illustration of the procedure used to build the IM.

In order to make the quantitative evaluation of several OF possible simultaneously from a global point of view, the OA indicator is proposed. It is obtained by following the procedure illustrated in Fig. 4: the IM rows are separated by a variable (Fig. 4, step1); for each variable, the average is taken along all wells (step2); the absolute value is taken (step3); and the variables are combined altogether by taking the average of their absolute values (step4). The OA can be analyzed in terms of each variable (oil and water production and Bottom-Hole Pressure (BHP) in this case) after step2 or in terms of all variables together (after step4). It permits the identification of attribute levels that may be causing high deviations as a whole.

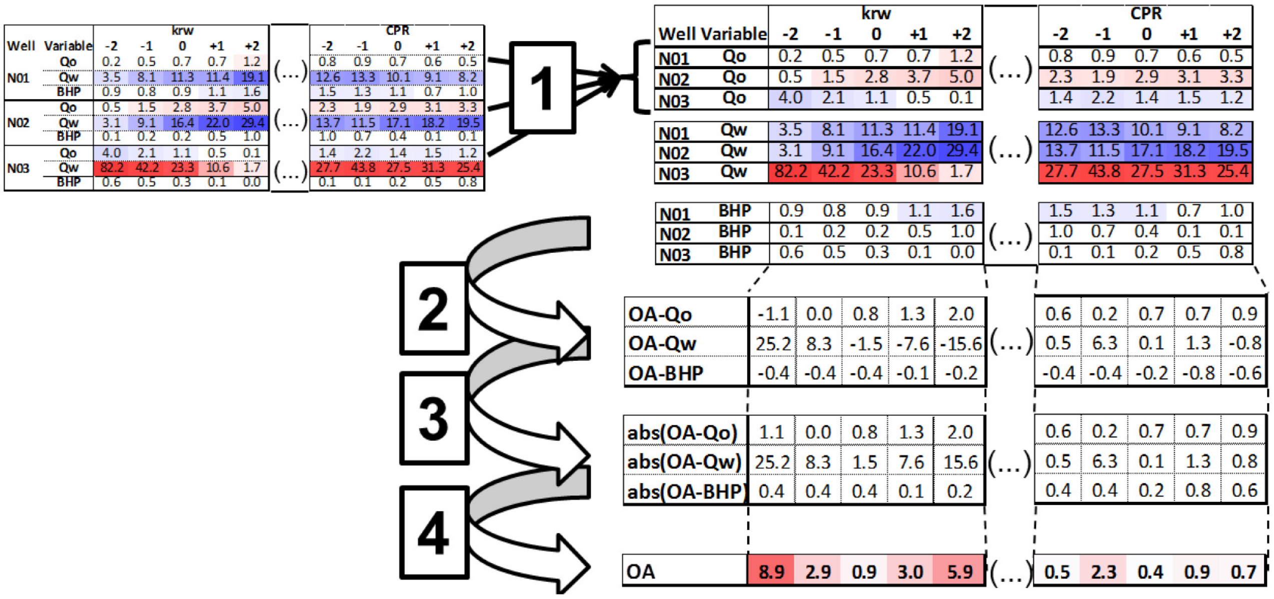

Figure 4—illustration of the procedure used to obtain the OA.

After obtaining the IM and the OA, they can be used for identifying evidences of the influence of uncertainties on the highest deviations. The analysis may focus on several OF from a global point of view

through the OA or on specific OF through the respective row in the IM. In both ways, the analysis should be performed for the attribute presenting the highest deviation and rely on the contrast among the levels of this attribute. Table 1 summarizes the guidelines to support the analysis of uncertainties influence and the possible remedial actions aiming to improve the history matching.

Table 1—guidelines for analysis of uncertainties influence.

Contrast from attribute levels	Are Images causing the highest deviations?	Highest deviation for the attribute	Remedial action
No contrast	-	All attribute levels are high	Model recharacterization:<br>- Modify images or<br>- input new uncertainty
High	No	Extreme level in the attribute	Uncertainties reduction: level exclusion
		Center level in the attribute	Model recharacterization: input new uncertainty
High	Yes	-	Model recharacterization: modify images

Comparison of data misfit among OF: Indexes

Indexes are proposed to highlight the most problematic OF and to evaluate the results along the iterations. The objective is to improve the visual comparison of matching quality through the NQDS plot (Almeida et al. 2014) and make it quantitative and systematic.

The matching quality of each OF is measured in two aspects: symmetry in relation to history, and deviation magnitude of NQDS values. These aspects are quantified by the symmetry and the deviation indexes, respectively:

$$SI = \frac{(n.of\ models\ with\ \boldsymbol{NQDS}>0)-(n.of\ models\ with\ \boldsymbol{NQDS}<0)}{N}, \tag{5}$$

$$DI = median[NQD_1, NQD_2, \ldots, NQD_N], \tag{6}$$

where:

SI = symmetry index;
N = total number of models generated through the DLHG method;
DI = deviation index;
NQD = absolute value of NQDS (Eq. 4).

Some OF may present a high absolute value of symmetry index (SI) and a low deviation index (DI) (for example, well 2 in Fig. 5) or, alternatively, a low SI absolute value and a high DI (for example, well 4 in Fig. 5). So, in order to make the comparison of the several OF and prioritization in local analysis easier, the total index (TI) is proposed, which simultaneously quantifies both symmetry and deviation magnitude aspects:

$$TI = \frac{SI}{|SI|} \times (|SI| + \alpha) \times DI, \qquad if\ SI \neq 0; \tag{7a}$$

$$TI = \alpha \times DI, \qquad if\ SI = 0. \tag{7b}$$

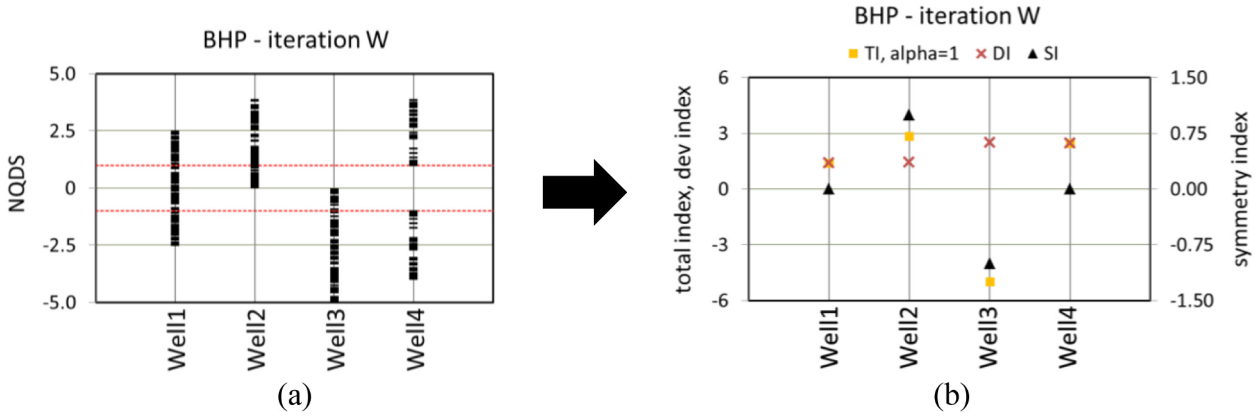

Figure 5—example of (a) NQDS results for a model realization set, and (b) respective indexes.

The α parameter is a non-negative number that should be adopted according to the desired importance for completely symmetric (SI=0) well variables. In this case, a high value for α will result in a relatively high TI and, if α is low, TI will be relatively low. The choice of a value higher than zero for α is recommended in order to prevent well variables similar to those of well 4, shown in Fig. 5a, from having TI=0 and, consequently, presenting a relatively good matching quality.

Total index should be interpreted as follows: the signal indicates whether more models are above (positive TI) or below (negative TI) the history data and the TI absolute value indicates how far the models' deviations are from history.

Model Filtering

The last step of the methodology is the generation of models to be employed in production prediction. At this stage, a reasonable number of models that pass through the established acceptance criteria is expected, i.e., their NQDS values are between −1 and +1 for all OF, simultaneously. For special cases in which this is difficult to obtain, the acceptance range may be weakened. The model realizations inside the acceptance criteria range are filtered and used as reservoir scenarios for production forecasting under uncertainties.

Application

The methodology is applied to the UNISIM-I-H model, which is a benchmark case for history matching and uncertainty reduction applications. The model is described in Avansi and Schiozer (2015). It is derived from the UNISIM-I model, a synthetic model based on real data from the Namorado field, located in the Campos basin, Brazil (Avansi and Schiozer 2015).

Fig. 6 shows the reservoir grid top including all 14 existent production wells (Fig. 6a) and all 11 existent water injection wells (Fig. 6b). There are two regions, which are isolated from each other through a fault with zero transmissibility, as indicated by the red arrow in Fig. 6.

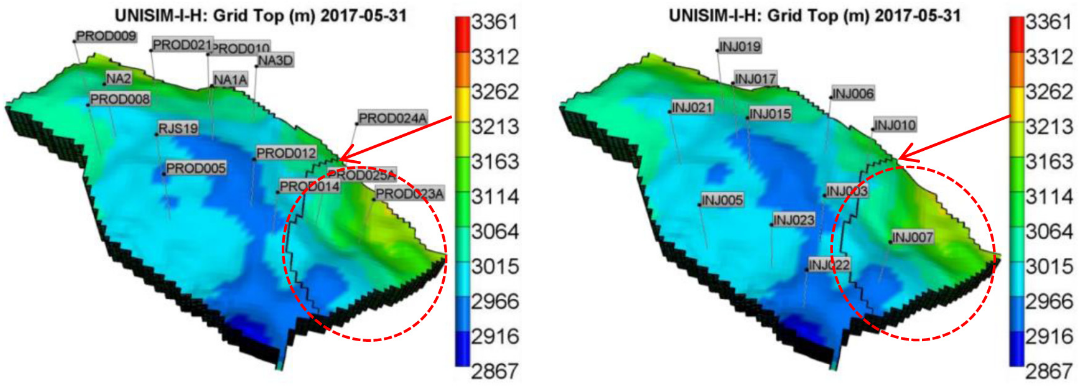

(a) (b)

Figure 6—grid top of the UNISIM-I-H model (Avansi and Schiozer 2015), including (a) all 14 production wells and (b) all injection wells. The sealing fault, as well as region 2, is indicated.

There are six original geological uncertainties, which are briefly described in Table 2. The relative permeability to water, petrophysical properties and PVT data for region 2 are defined in discrete form from the model characterization phase and are considered in this work at all attribute levels. The three other uncertain attributes (initial water-oil contact (WOC) depth for region 2, vertical permeability multiplier and rock compressibility) are in continuous form, and were discretized from the probability density function (originated at the model characterization phase). The number of levels employed is shown in the last column of Table 2.

Table 2—description of original geological uncertainties.

Nomenclature	Description	Number of levels
k_{rw}	Relative permeability to water, comprised of the relative permeability curve for the water and oil phases	5
petrophysical properties	Porosity, horizontal and vertical permeabilities and net to gross, generated from the Sequential Gaussian Simulation technique applied to porosity property, while the other properties were correlated to porosity from specific parameters applied to a spherical variogram model	500
PVT_{r2}	PVT data for region 2, which present different fluid properties for solution gas-oil ratio (R_s), formation-volume factor for oil (B_o) and for gas (B_g), oil (μ_o) and gas viscosity (μ_g) and oil compressibility (c_o)	3
WOC_{r2}	Initial WOC depth for region 2	5
mKz	Vertical permeability multiplier	5
CPR	Rock compressibility	5

The production history is comprised of fluid rates (water production rate, oil production rate, and gas production rate from all 14 producers and the water injection rate from all 11 injectors) and BHP from all wells. Tol and C values (Eq. 3) employed to normalize the deviations in the history matching procedure are shown in Table 3 for each history data series. The variables informed to the simulator are liquids production rate (Q_l) for production wells, and water injection rate (Q_{wi}) for injection wells.

Table 3—values adopted to normalize the deviations.

	Production wells					Injection wells	
	Q_l	Q_o	Q_w	Q_g	BHP	Q_{wi}	BHP
Tol	2.5%	10%	10%	20%	10%	2.5%	10%
C (m^3/d)	0	0	20	0	0	0	0

In this work, when petrophysical images are indicated to be the cause of high deviations and, thus, modifications to them are necessary, a procedure for the history matching diagnosis and application of changes to the images is employed. Firstly, a region comprising the grid blocks is defined based on the diagnosis of the well presenting high deviation through streamlines visualization for a base model. After that, the petrophysical properties in this region are modified by applying coefficients of multiplication and sum operation to the old properties' values. The coefficient values are tested in an attempt to produce results close to the established objective in diagnosis for the whole set of model realizations. A range of coefficient values associated to a probability of occurrence is then established and incorporated into the model as new uncertainty.

Results

All the uncertainties described in Table 2 were sampled and combined to generate 500 model realizations through discretized Latin Hypercube with geostatistics. A previous work (Schiozer et al. 2015) demonstrated that 100 to 200 petrophysical images are sufficient to represent the petrophysical properties' distribution for the UNISIM-I-H for history matching purposes. Based on that, and in order to reduce the number of images to be used in the procedure, 100 images were selected based on the lowest sum of normalized quadratic deviation with sign of Q_l and Q_{wi} from all wells. Q_l and Q_{wi} were used as criteria to select the images because they are the variables informed to the simulator and were not being honored for all model realizations.

In the first iteration, 100 model realizations were generated through DLHG by employing the original model uncertainties (Table 2) and the 100 images previously selected. In order to verify the data misfit of these new models, the distribution of models by maximum |NQDS| among all OF is evaluated.

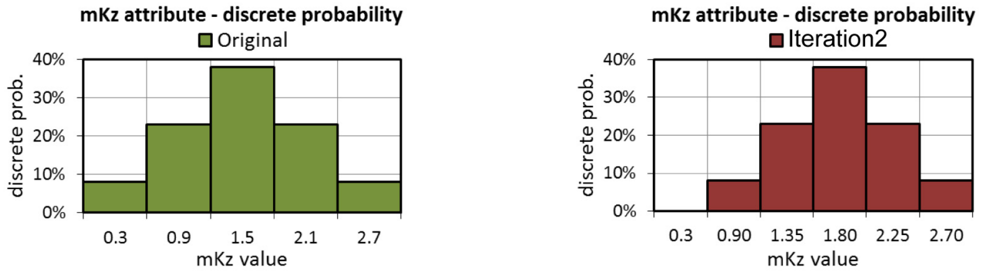

(a) (b)

Figure 7—mKz attribute levels (a) before and (b) after the modification implemented in iteration 2.

It can be seen in Fig. 8 that no model can pass through the desired acceptance criteria, i.e., no model presents |NQDS|≤1 for all well variables simultaneously. Also, one model passes through an acceptance criteria just for |NQDS|≤20, showing the necessity to start the matching procedure.

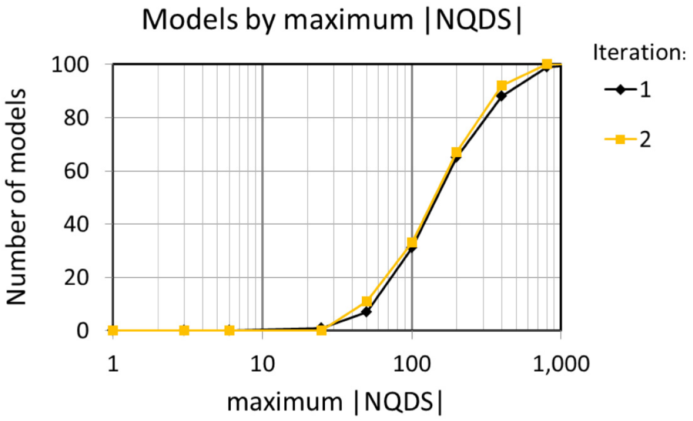

Figure 8—distribution of models by maximum |NQDS| among all OF, iteration 1 and 2.

The influence analysis of the uncertainties was performed in iteration 1, using the IM and focusing on attributes that have an influence on all OF. Thus, the OA was considered by taking all variables together. Results are shown in Table 4. The vertical permeability multiplier (mKz) presents the highest value among all uncertain attributes. This is evidence that mKz is the most influential uncertainty of the high deviations from a global point of view. In addition, the mKz level at which the highest value occurs shows a high contrast compared to the other levels, as highlighted in Table 4 and, thus, indicates that it is the cause of the highest deviations as a whole.

Table 4—OA and modifications performed from iterations 1 to 9.

		Attribute level																							
Iteration		PVT_r2			krw					mKz					CPR					WOC_r2					Indicated action (level elimination)
1		-1	0	+1	-2	-1	0	+1	+2	-2	-1	0	+1	+2	-2	-1	0	+1	+2	-2	-1	0	+1	+2	mKz -2 level
	OA	2	2.1	2.2	3.1	2.1	1.6	4.1	7.2	8.4	2.6	1.7	1.4	1.2	1.6	2.8	2.5	1.5	3.2	2.1	2.1	2.4	2.2	2.4	
2	OA	-1	0	+1	-2	-1	0	+1	+2	-2a	-1a	0a	+1a	+2	-2	-1	0	+1	+2	-2	-1	0	+1	+2	krw +2 level
		1.8	1.7	1.8	3.9	1.9	1.2	2.9	6.3	5.7	1.5	1.6	2.1	0.9	1.8	1.6	1.7	3.1	1.4	2.1	1.7	1.7	1.6	1.5	
3	OA	-1	0	+1	-2	-1a	0a	+1a	+2a	-2a	-1a	0a	+1a	+2	-2	-1	0	+1	+2	-2	-1	0	+1	+2	krw -2 level
		2.1	1.7	1.9	4.3	2.6	1.9	1.6	2.7	1.8	2	1.6	2.4	2.7	1.9	2.5	1.9	1.3	2	1.9	1.6	1.8	1.8	3.6	
4	OA	-1	0	+1	-2a	-1b	0b	+1b	+2a	-2a	-1a	0a	+1a	+2	-2	-1	0	+1	+2	-2	-1	0	+1	+2	krw -2 level
	(Qw)	1	0.4	6.3	12.2	8	2.7	-1.2	-8.8	-3.4	-0.9	3.7	3.9	9.6	4.5	-0.5	0.9	5.1	10.3	7.9	1.4	1.5	4.3	1.0	
5	OA	-1	0	+1	-2b	-1c	0c	+1c	+2a	-2a	-1a	0a	+1a	+2	-2	-1	0	+1	+2	-2	-1	0	+1	+2	krw +2 level
		1.2	0.9	1	1.8	1.5	1.1	2	2.9	1.7	1.9	1.1	1.6	1.2	1.3	1.4	1.3	1.1	1.8	0.8	1	1.2	2.1	1.2	
6	OA	-1	0	+1	-2b	-1d	0d	+1d	+2b	-2a	-1a	0a	+1a	+2	-2	-1	0	+1	+2	-2	-1	0	+1	+2	WOC_r2 -2 level
	(BHP)	2	1.9	2.6	1.2	1.3	2.2	1.5	4.6	3	3.3	2	1.4	1.1	1.4	2.3	1.8	2.6	3.1	5	1.8	2.4	1.6	0.8	
7	OA	-1	0	+1	-2b	-1d	0d	+1d	+2b	-2a	-1a	0a	+1a	+2	-2	-1	0	+1	+2	-2a	-1a	0a	+1a	+2	krw -2 level
		1.4	1.2	0.9	2.5	1.1	1	1.3	1.4	1.9	1	1.2	1.2	1.5	0.9	0.9	1.1	1.2	1.8	1.1	1	1.1	1.3	1.0	
8	OA	-1	0	+1	-2c	-1e	0e	+1e	+2b	-2a	-1a	0a	+1a	+2	-2	-1	0	+1	+2	-2a	-1a	0a	+1a	+2	mKz -2 level
		1.2	1	1.2	1.2	1.2	1.1	1.3	2	2.4	1	1.1	0.8	1.1	0.8	1.3	1	1	1.4	1.3	1	1.0	1.2	1.4	
9	OA	-1	0	+1	-2c	-1e	0e	+1e	+2b	-2b	-1b	0b	+1b	+2	-2	-1	0	+1	+2	-2a	-1a	0a	+1a	+2	CPR +2 level
	(BHP)	1.5	2.5	1.6	1.8	1.8	2	1.8	1.9	2.3	2	1.8	1.7	1.4	-0.7	1.9	1.8	2.2	3.6	1.8	1.6	2.0	1.8	2.0	

Besides the strong contrast among mKz levels, the highest OA value occurs at an extreme level (mKz -2 level), so the indicated modification is the level exclusion, as shown in Fig. 7.

Iteration 2 was performed by simulating a new DLHG combination (100 models), now with the new mKz distribution (Fig. 7b). Fig. 8 shows the comparison of results between iteration 1 and 2 in terms of

the distribution of models by maximum |NQDS| among all OF. Although small, an improvement can be seen for some ranges.

From iteration 2 to 9, the influence analysis from uncertainties on high deviations continued focusing on attributes with global effect on the several OF. At each iteration, the OA was analyzed, first for all variables together, as performed in iteration 1. When no evidence of high deviations could be found, it progressed to the analysis of OA by variable, by prioritizing the ones with the worst matching for the wells. Table 4 shows the OA that evidenced the level exclusion considered at each iteration. Each time an uncertain attribute is modified, the changed attribute levels receive a letter (for example, level +2b indicates that level +2 was modified twice in relation to the original model uncertainties).

At iteration 10, there was no uncertainty presenting a high contrast among the high OA values or, in other words, no evidence of global influence from uncertainties on high deviations was encountered. At this point, the analysis of uncertainties influence progressed to the local focus by analyzing the individual OF with the worst matching through the TI.

Fig. 9 presents the TI and the NQDS at iteration 10 for (a) water production rate, (b) BHP from all producers and (c) BHP from all injectors. Q_l and Q_{wi} are being honored for all wells in practically all model realizations (TI equals zero); and the NQDS for gas production rate keeps a perfect correlation with the oil production rate for all producers. Therefore, these well variables are not shown here, and do not need to be evaluated.

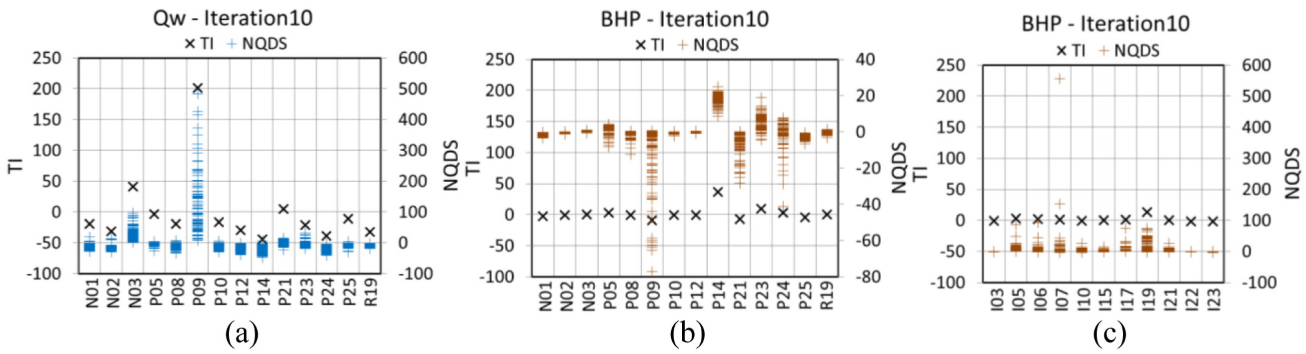

Figure 9—TI and NQDS for iteration 10: (a) water production rate, (b) BHP for all producers and (c) BHP for all injectors.

TI for PROD09 Q_w (P09 in Fig. 9a) presents the highest absolute value among all well variables, so it is the OF with the worst matching quality. At this point, the influence from uncertainties on PROD09 is evaluated through the IM (Table 5).

Table 5—IM for PROD09 variables, iteration 10.

	PVT_{r2}			k_{rw}					mKz					CPR					WOC_{r2}				
Variable	-1	0	+1	-2c	-1e	0e	+1e	+2b	-2b	-1b	0b	+1b	+2	-2	-1a	0a	+1a	+2a	-2a	-1a	0a	+1a	+2
Qo	6	7	4	7	6	5	4	4	4	6	5	5	6	5	7	6	5	5	8	5	5	6	6
Qw	134	150	96	171	147	115	99	102	92	144	123	123	144	113	148	129	114	104	180	109	121	132	139
Qg	1	2	1	2	2	1	1	1	1	2	1	1	2	1	2	1	1	1	2	1	1	1	1
QL	1	0	0	0	0	1	0	0	2	0	0	0	0	0	1	0	0	0	0	0	0	1	0
BHP	17	12	10	12	14	11	12	18	23	19	13	5	14	21	19	11	12	4	14	8	15	17	8

The highest IM value of PROD09 Q_w occurs for WOC_{r2} -2a level. Although it occurs at an extreme level of the attribute, there is no strong contrast with the other WOC_{r2} levels, so there is no evidence of

high deviations caused by the uncertain attributes through the IM. An analysis of petrophysical images confirms that they all present high deviations for PROD09 Q_w and, thus, the images need to be modified.

The diagnosis of PROD09 Q_w through analysis of the production curves (Fig. 10a) was employed to implement changes to the images. The procedure to modify the petrophysical images as described in the application section of this work was applied to achieve the objective. This involved the (a) analysis of other producers in PROD09 area, (b) selection of some models to be tested during the modifications, (c) identification of a water source through streamlines visualization, (d) definition of a region to have the properties changed by coefficients, and (e) establishment of a range of coefficient values associated to a probability of occurrence to be incorporated as a new uncertainty in the model.

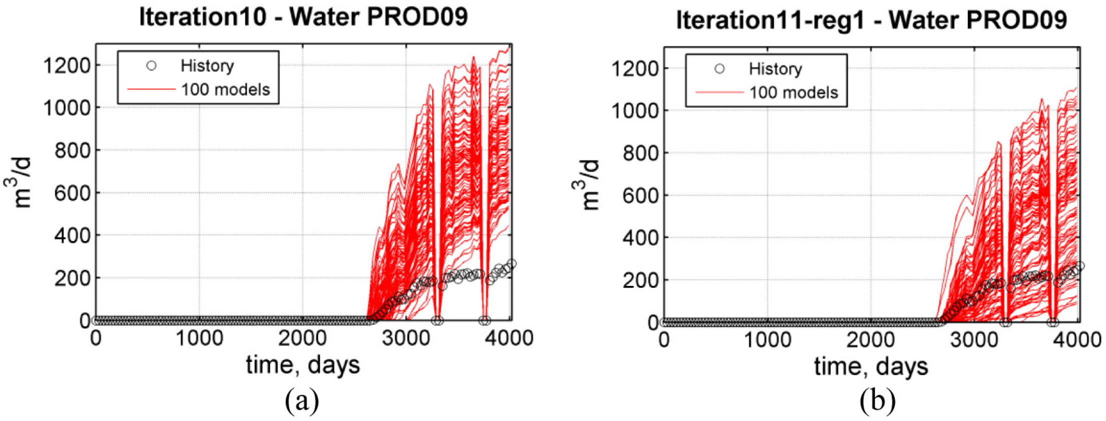

Figure 10—water production curves for PROD09 Q_w (a) before the change to images and (b) after the applied change.

After applying the procedure, a new iteration was performed by incorporating a coefficient to reduce the permeability in the defined region of grid blocks and, consequently, the water flow from the diagnosed water source to PROD09 as a new uncertainty. Results are presented in Fig. 10b, which evidences a good improvement in water production for PROD09.

From iteration 11 to 15, the influence analysis from uncertainties on high deviations continued focusing on the individual OF, by prioritizing the ones with worst matching quality (highest absolute value of TI among all OF). When diagnosis indicated that water production was the problem to be tackled, the images were modified by following the same procedure as applied to PROD09 Q_w in iteration 10. At iteration 14, the well variable that presented the worst matching was PROD14 BHP, which had high NQDS values for all model realizations. Q_w of PROD14 had a good history matching quality and another completed producer very close to it had both Q_w and BHP with good matching as well. These two aspects led to the conclusion that the distributions of petrophysical properties were probably not the cause of PROD14 BHP high deviations, and the decision taken was the productivity adjustment. Table 6 shows a summary of the diagnosed OF and analysis performed from iteration 10 to 15.

Table 6—analysis performed for OF from iteration 10 to 15.

Iteration	Diagnosed OF	Analysis
10	PROD09 Q_w	Simulated Q_w was higher than history
11	PROD14 Q_w	Simulated water breakthrough was delayed and Q_w was lower in relation to the history
12	PROD24 Q_w	Simulated water breakthrough was delayed and Q_w was lower in relation to the history
13	NA3D Q_w	Simulated water breakthrough was anticipated and Q_w was higher in relation to the history
14	PROD14 BHP	Simulated BHP was higher than history
15	RJS19 Q_w	Simulated water breakthrough was delayed and Q_w was lower in relation to the history

After the modification implemented in iteration 15, a new DLHG was generated and the model combinations simulated in iteration 16. Fig. 11 presents the evaluation of history matching improvement on individual OF through TI, from iteration 1 to 16. During the modifications that focused on uncertainties with global effect (iteration 1 to 10), BHP of injection wells was the most improved OF, while producers were not highly affected in general.

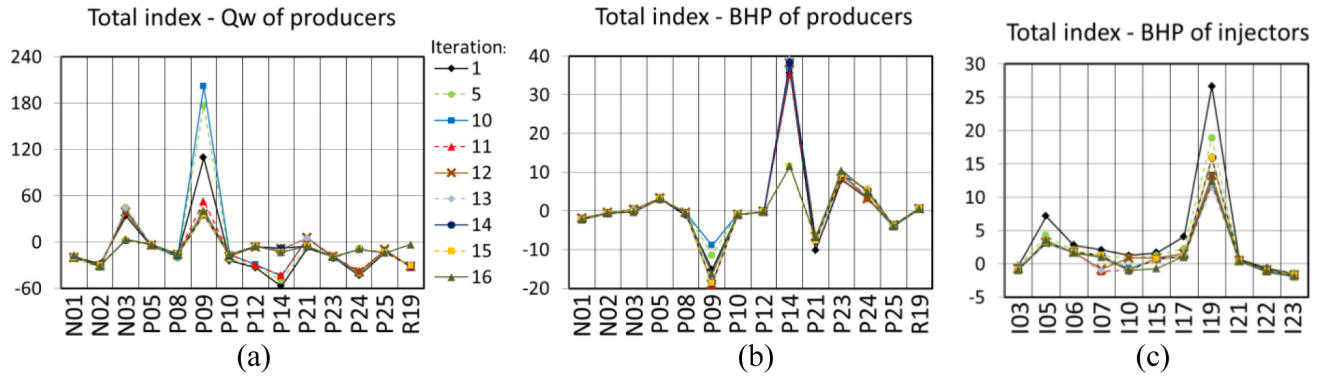

Figure 11—TI along the applied model modifications: (a) water production rate, (b) BHP for all producers and (c) BHP for all injectors.

The substantial improvement in specific OF was obtained along the local modifications (Table 6), each being reflected in TI (Fig. 11): PROD09 Q_w improved from 201.7 (iteration 10) to 52.9 (iteration 11), PROD14 Q_w from −42.9 (iteration 11) to −9.3 (iteration 12), PROD24 Q_w from −39.1 (iteration 12) to −9.0 (iteration 13), NA3D Q_w from 43.7 (iteration 13) to 2.8 (iteration 14), PROD14 BHP from 38.4 (iteration 14) to 11.5 (iteration 15) and RJS19 Q_w from −31.0 (iteration 15) to −3.1 (iteration 16).

Although no model can yet pass through the acceptance criteria for all OF (|NQDS|≤1), a good improvement can be noticed along the modifications, especially on the highest deviations range, for both global analysis (from iteration 1 to 10) and local analysis phase (from iteration 10 to 16) (Fig. 12).

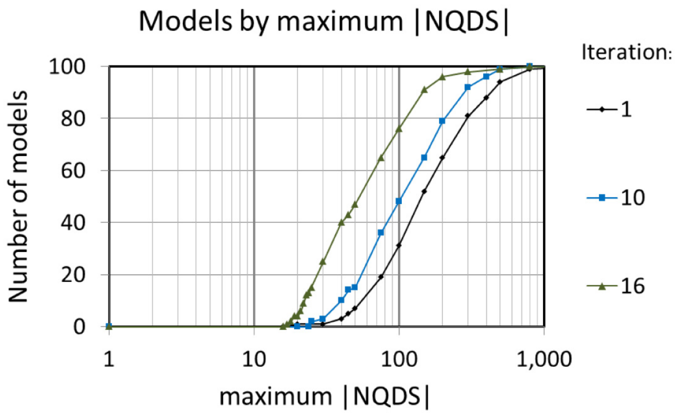

Figure 12—distribution of models by maximum |NQDS| among all OF, throughout the applied model modifications.

At this point it is believed that the difficulty for obtaining models that honor all the well history data is caused by some individual OF with low number of models within the acceptance range. Fig. 13 presents an analysis assuming less strict ranges, i.e., it shows the number of models with |NQDS|<5 and |NQDS|<25 for each individual OF. Similar to the TI analysis (Fig. 11) that evidenced the good improvements achieved along the local modifications, here the same can be concluded. However,

important problems are still faced, especially with Q_w of some producers that were not targeted along the local changes (N01, N02, P08, P10 and P23 wells). Thus, the application to the UNISIM-I-H model will continue to progress and results including the model filtering phase and production forecasting under uncertainties will be published in the future.

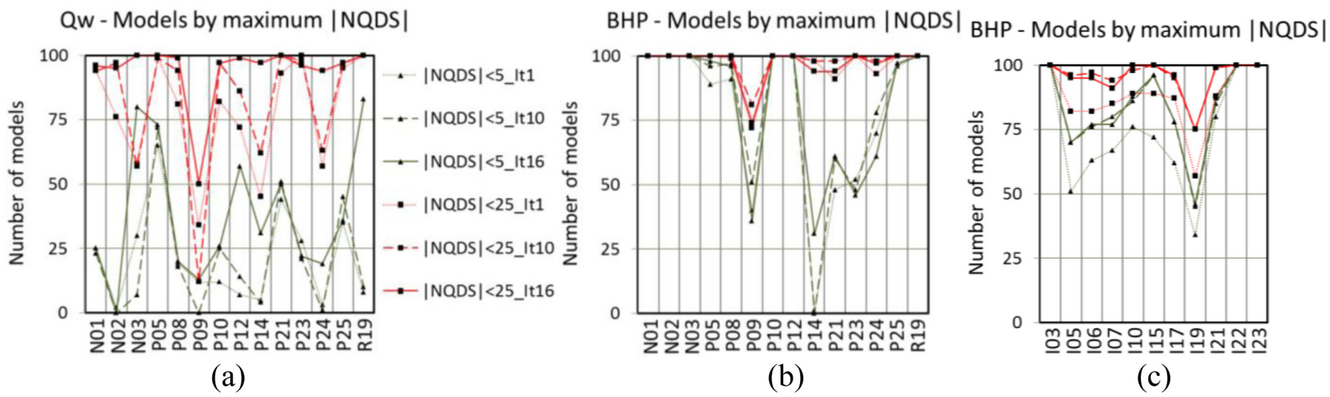

Figure 13—number of models with |NQDS|<5 and <25 for each individual OF: (a) Q_w, (b) BHP of producers and (c) BHP of injectors.

Conclusions

- The developed indicators (influence matrix, overall average and total index) provide a systematic framework to reduce reservoir uncertainties through history matching in a multi-objective approach. They support gradual modifications to the models' uncertainties by evidencing the source of the highest deviations from history at both global and local levels. This feature allows prioritizing model modifications in a way that provides robustness to the process.
- The use of the proposed tools facilitates the application to complex cases, which involve data available from several wells and many geological uncertainties, because they provide a systematic means to analyze the models' deviations simultaneously for all objective functions and all uncertain attributes.
- The IM and the derived OA indicators were created to support the analysis and identification of models with the highest deviations caused by uncertain properties on a quantitative basis. The analysis may be carried out at a global level through the OA, which focuses on deviations for various OF simultaneously, or at a local level, focusing on an individual case directly through the IM rows.
- The identification of the cause of high deviations, through the use of IM and OA, relies on the contrast among the different uncertain attributes and has to be performed carefully. Although they are quantitative, a minimum value must be chosen to decide whether the contrast among uncertain attribute levels is sufficient or not to tag an attribute as the cause of high deviations and must be established by the professional conducting the process.
- TI is an indicator created with the purpose of representing, for each OF, the history matching quality of the whole set of model realizations. It combines the key aspects of models' deviations from the NQDS plot: the symmetry and the magnitude of the deviations in relation to the history data. One advantage of TI use in the process is that it may be easily incorporated into an automatic algorithm to prioritize model modifications and achieve specific targets when a desired criterion is established.
- The application to the UNISIM-I-H model demonstrated satisfactorily how the developed indicators may be applied to support model modifications in an iterative procedure to reduce reservoir uncertainties based on evidences of high deviations from history data.

- The next step of the work will be to progress on the application to the UNISIM-I-H model, aiming to achieve the final desired criteria on history matching quality for all OF (|NQDS|<1) and then to perform production forecasting under uncertainties.

Nomenclature

α	=	*non-negative number*
AQD	=	*acceptable quadratic deviation*
Bg	=	*formation-volume factor for gas,* m^3/m^3
Bo	=	*formation-volume factor for oil,* m^3/m^3
c_o	=	*oil compressibility,* $m^3/kg/cm^2$
R_s	=	*solution gas-oil ratio,* m^3/m^3
μ_o	=	*oil viscosity, mPa-s*
μ_g	=	*gas viscosity, mPa-s*
BHP	=	*bottom-hole pressure,* kg/cm^2
C	=	*positive constant*
CPR	=	*rock compressibility,* $m^3/kg/cm^2$
DI	=	*deviation index*
$Hist_i$	=	*history data value at time point "i"*
LD	=	*linear deviation of a simulated data series from a history data series*
IM	=	*influence matrix*
k_{rw}	=	*relative permeability to water*
mKz	=	*vertical permeability multiplier*
n	=	*number of points for the respective data series*
N	=	*number of model realizations*
NQD	=	*absolute value of a normalized quadratic deviation with sign*
NQDS	=	*normalized quadratic deviation with sign*
OA	=	*overall average*
QDS	=	*quadratic deviation with sign of a simulated data series from a history data series*
Q_g	=	*gas production rate,* m^3/d
Q_l	=	*liquid production rate,* m^3/d
Q_o	=	*oil production rate,* m^3/d
Q_w	=	*water production rate,* m^3/d
Q_{wi}	=	*water injection rate,* m^3/d
SI	=	*symmetry index*
Sim_i	=	*simulated value at time point "i" for the data series*
TI	=	*total index*
Tol	=	*acceptable tolerance*

Acknowledgments

The authors thank UNISIM, CEPETRO, Division of Petroleum Engineering (DEP-FEM), UNICAMP, BG Group and PRH-ANP/MCTI for their support in the development of this work and the Computer Modelling Group (CMG) for the software licenses.

References

Abdollahzadeh, A., Reynolds, A., Christie, M., Corne, D., Davies, B. and Williams, G. 2011. Bayesian optimization algorithm applied to uncertainty quantification. Presented at the SPE Europec/EAGE annual conference and exhibition, Vienna. SPE-143290-MS. http://dx.doi.org/10.2118/143290-MS.

Al-Shamma, B.R. and Teigland, R. 2006. History matching of the Valhall field using a global optimization method and uncertainty assessment. Presented at the SPE Annual Technical Conference and Exhibition, San Antonio. SPE-100946-MS. http://dx.doi.org/10.2118/100946-MS.

Almeida, F.L.R., Davolio, A. and Schiozer, D.J. 2014. A New Approach to Perform a Probabilistic and Multi-objective History Matching. Presented at the SPE Annual Technical Conference and Exhibition, Amsterdam, 27-29 October, SPE-170623-MS. http://dx.doi.org/10.2118/170623-MS.

Avansi, G.D. and Schiozer, D.J. 2015. A New Approach to History Matching Using Reservoir Characterization and Reservoir Simulation Integrated Studies. Prepared for presentation at the Offshore Technology Conference to be held in Houston, 4-7 May. Paper OTC-26038-MS.

Becerra, G.G., Maschio, C. and Schiozer, D.J. 2008. Uncertainties Mitigation through Integration with Production History Matching. Presented at the Rio Oil & Gas Expo and Conference, Rio, 15-18 September. (In Portuguese).

Becerra, G.G., Maschio, C. and Schiozer, D.J. 2011. Petroleum Reservoir Uncertainty Mitigation through the Integration with Production History Matching. *J. of the Braz. Soc. of Mech. Sci. & Eng.* **33**(2): 147–158.

Hajizadeh, Y., Christie, M. and Demyanov, V. 2011. Ant colony optimization for history matching and uncertainty quantification of reservoir models. *J. Pet. Sci. Eng.* **77**(1): 78–92.

Li, G. and Reynolds, A.C. 2011. Uncertainty quantification of reservoir performance predictions using a stochastic optimization algorithm. *Comput. Geosci.* **15**: 451–462.

Martínez, J.L.F., Mukerji, T., Gonzalo, E.G. and Suman, A. 2012. Reservoir characterization and inversion uncertainty via a family of particle swarm optimizers. *Geophysics* **77**: 1–16.

Maschio, C., Schiozer, D.J. and Moura, Filho, M.A.B. 2005. A Methodology to Quantify the Impact of Uncertainties in the History Matching Process. Presented at the SPE Annual Technical Conference and Exhibition, Dallas, 9-12 October. SPE-96613-MS. http://dx.doi.org/10.2118/96613-MS.

Maschio, C., Schiozer, D.J., Moura, Filho, M.A.B. and Becerra, G.G. 2009. A Methodology to Reduce Uncertainty Constrained to Observed Data. *SPE Res. Eval. & Eng.* 167–180.

Maschio, C., Carvalho, C.P.V. and Schiozer, D.J. 2010. A New Methodology to Reduce Uncertainties in Reservoir Simulation Models Using Observed Data and Sampling Techniques. *J. Pet. Sci. Eng.* **72**: 110–119.

Risso, F.V.A., Risso, V.F. and Schiozer, D.J. 2011. Risk Analysis of Petroleum Fields Using Latin Hypercube, Monte Carlo and Derivative Tree Techniques. *Journal of Petroleum and Gas Exploration Research* **1**(1): 14–21.

Schiozer, D.J., Avansi, G.D. and Santos, A.A.S. In press. A New Methodology for Risk Quantification Combining Geostatistical Realizations and Discretized Latin Hypercube. *J. Pet. Sci. Eng.* (submitted 09 January 2015).
